# Lack of the α1,3-Fucosyltransferase Gene (*Osfuct*) Affects Anther Development and Pollen Viability in Rice

**DOI:** 10.3390/ijms19041225

**Published:** 2018-04-18

**Authors:** Joon-Soo Sim, Mahipal Singh Kesawat, Manu Kumar, Su-Yeon Kim, Vimalraj Mani, Parthiban Subramanian, Soyoung Park, Chang-Muk Lee, Seong-Ryong Kim, Bum-Soo Hahn

**Affiliations:** 1 Metabolic Engineering Division, Department of Agricultural Biotechnology, National Institute of Agricultural Sciences, Rural Development Administration, Jeonju 54874, Korea; jssim@korea.kr (J.-S.S.); mahibiotech@snu.ac.kr (M.S.K.); suyeon4617@gmail.com (S.-Y.K.); vimal@jbnu.ac.kr (V.M.); parthi@chungbuk.ac.kr (P.S.); psy0203@korea.kr (S.P.); changmuk@korea.kr (C.-M.L.); 2 Department of Life Sciences, Sogang University, Seoul 121-742, Korea; manukumar007@gmail.com (M.K.); sungkim@sogang.ac.kr (S.-R.K.)

**Keywords:** α1,3-fucosyltransferase, anther, development, pollen, microarray, *N*-glycan, viability

## Abstract

*N*-linked glycosylation is one of the key post-translational modifications. α1,3-Fucosyltransferase (OsFucT) is responsible for transferring α1,3-linked fucose residues to the glycoprotein *N*-glycan in plants. We characterized an *Osfuct* mutant that displayed pleiotropic developmental defects, such as impaired anther and pollen development, diminished growth, shorter plant height, fewer tillers, and shorter panicle length and internodes under field conditions. In addition, the anthers were curved, the pollen grains were shriveled, and pollen viability and pollen number per anther decreased dramatically in the mutant. Matrix-assisted laser desorption/ionization time-of-flight analyses of the *N*-glycans revealed that α1,3-fucose was lacking in the *N*-glycan structure of the mutant. Mutant complementation revealed that the phenotype was caused by loss of *Osfuct* function. Transcriptome profiling also showed that several genes essential for plant developmental processes were significantly altered in the mutant, including protein kinases, transcription factors, genes involved in metabolism, genes related to protein synthesis, and hypothetical proteins. Moreover, the mutant exhibited sensitivity to an increased concentration of salt. This study facilitates a further understanding of the function of genes mediating *N*-glycan modification and anther and pollen development in rice.

## 1. Introduction

*N*-glycosylation is a key post-translational modification that regulates the function of numerous proteins. *N*-glycoproteins have been implicated in diverse biological processes, such as protein folding, stability, protein–protein interactions, immune defense, inflammation, fertilization, embryogenesis and development; and also cause many diseases, including type 1 diabetes, Crohn’s disease, rheumatoid arthritis, cancers, and neurological disorders [1,2]. *N*-glycoproteins alter the functions of numerous cell-surface proteins participating in adhesion and migration [3]. The initial *N*-glycosylation process occurs on the luminal side of the endoplasmic reticulum, where an oligosaccharide is assembled on the lipid carrier dolichylpyrophosphate and is transferred to the asparagine residue in a nascent polypeptide [4]. The primary glycan structure is composed of two *N*-acetyl glucosamine and three mannose residues, and this core glycan is elaborated and modified further, resulting in a wide range of *N*-glycan structures. Subsequent processing and modification of the oligosaccharide chain takes place in the Golgi complex and synthesis of *N*-linked glycan ends at the trans-Golgi network to generate the high structural diversity of *N*-linked glycans in a cell-type and species-specific manner [5]. Although the first step in the formation of the oligomannosidic structure in the *N*-glycosylation pathway is conserved in all domains of life, the final complex step of *N*-glycan synthesis differs and requires the activities of several specific glycosyltransferases [2]. The paucimannosidic *N*-glycan with core β1,2-xylose and α1,3-fucose residues (PNGXF; Man_3_XylFucGlcNAc_2_) is the most abundant *N*-glycan in plants [6]; however, these *N*-linked glycans are not present in mammals and constitute epitopes for carbohydrate-reactive antibodies that cause allergic reactions. In addition, Lewis A-containing glycoproteins also contribute to antigenicity [7]. Several genes implicated in *N*-glycan biosynthesis have been identified and characterized in plants [2]. The *Osdgl1* mutant modulates root cell wall polysaccharide composition, a smaller root meristem, shorter root cells, and death of root cells [8]. *SETH1* and *SETH2* are involved in the first step of the glycosylphosphatidylinositol biosynthetic pathway. A defect in *SETH1* and *SETH2* affects pollen germination, tube growth, and male transmission in *Arabidopsis* [9]. The *Arabidopsis* T-DNA insertion mutant (*alg10-1*) encoding alpha1, 2-glucosyltransferase exhibits a severe *N*-glycosylation defect leading to leaf growth and increased salt sensitivity in *Arabidopsis* [10]. Proper maturation of *N*-glycans in the Golgi complex is essential for salt tolerance in *Arabidopsis*. The defect in *N*-glycan synthesis, processing, and maturation of complex glycan 1 (*cgl1*) are more salt-sensitive compared with the wild type [11]. Strasser et al. [12] generated knockout *Arabidopsis* plants missing the β1,2-xylose and α1,3-fucose residues from complex *N*-glycans. The double knockout of α1,3-fucosyltransferase and β1,2-xylosyltransferase was reported in *Physcomitrella patens*. The single and double knockout lack the endogenous transcript of corresponding genes and do not differ in morphology, growth, development, or the ability to secrete a recombinant protein compared with wild-type moss [13]. α1,3-Fucosyltransferase and β1,2-xylosyltransferase are deactivated by multiplex CRISPR/Cas9 in *Nicotiana tabacum* BY-2 cells that produce glycoproteins deficient in plant-specific *N*-glycans. The knockout lines differ in morphology from the wild type [14]. Two rice mutants (*fuct-1* and *fuct-2*) with loss of *Osfuct* function also display larger tiller angles, shorter panicle lengths and internodes, reduced grain filling, and an increase in the number of unusual-shaped chalky grains under greenhouse conditions. The *fuct-1* and *fuct-2* mutants also show decreased gravitropic responses. The *fuct-1* and *fuct-2* mutations affect basipetal auxin transport and accumulation, resulting in a decreased gravitropic response in rice [15]. Although the synthesis, processing, and maturation of *N*-glycosylation are well investigated in animals, the underlying molecular mechanism is poorly understood in plants. It has been hypothesized that complex *N*-glycoproteins are not required for plant growth and developmental processes.

In this study, we characterized a T-DNA-inserted mutant *Osfuct*, by knocking out the α1,3-fucosyltransferase (*OsFucT*) gene in rice. The mutant exhibited defects in anther and pollen development. The pollen grains of the mutant were shriveled and significantly smaller in size. Furthermore, the number of pollen grains per anther and viability decreased dramatically in the mutant compared to the wild type. The mutant was shorter, with fewer tillers, and had shorter internode and panicle lengths under field conditions. Our results revealed that the mutant produces *N*-glycans lacking the core α1,3-fucose residue. Genetic complementation analyses also revealed that rescue lines exhibited normal morphology and development; hence, a mutation in the intron region of the *Osfuct* gene was responsible for the pleiotropic phenotypes and defects in anther development. Our microarray data also revealed that several genes essential in plant developmental processes were significantly altered in the mutant. Therefore, these results suggest that α1,3-fucosyltransferase plays a fundamental role in plant growth and reproductive development.

## 2. Results

### 2.1. Identification and Isolation of a T-DNA Inserted Allele in the Rice Osfuct Gene 

**Figure 1:**
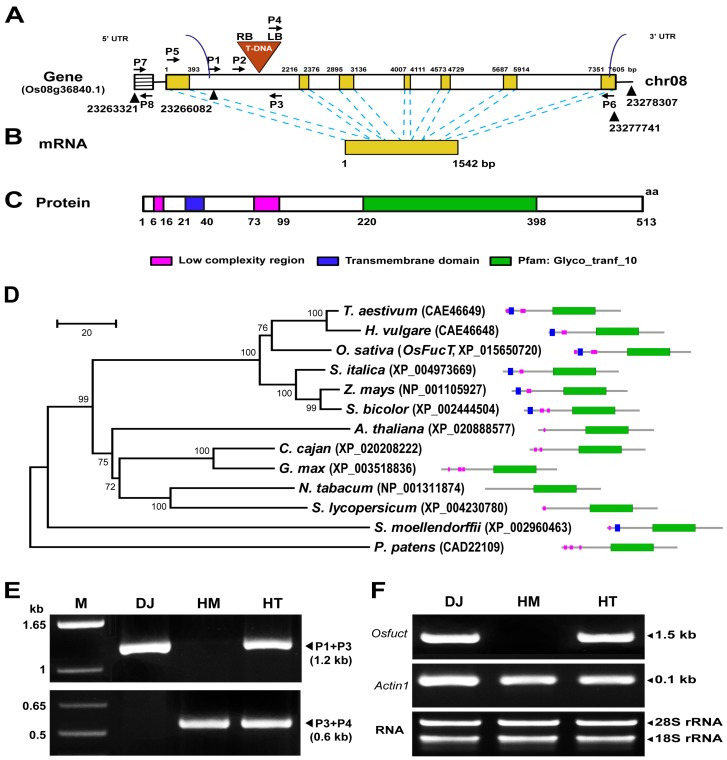
Identification and genotyping of the T-DNA insertion *Osfuct* mutant in rice (*Oryza sativa*). (**A**) Schematic representation of the genomic structure of the *Osfuct* and T-DNA insertion sites. Black-lined box represents promotor, while the yellow box indicates exons, and the white box represents the introns. Locations of the T-DNA insertion and direction of the left and right borders are indicated by an inverted triangle. Gene-specific (P1, P2, and P3) and T-DNA border sequence primers (P4) were used for genotyping and gene-specific primers (P5 and P6) were used for reverse transcription polymerase chain reaction (RT-PCR) analysis as indicated by the black arrowhead. (**B**) Yellow box with black border represents the full length of the cDNA (1542 bp). (**C**) SMART annotation for the OsFucT protein. Magenta box indicates the low complexity region, blue box shows transmembrane domain, and the green box represents the conserved glycosyltransferase domain (220–398 amino acids). (**D**) Phylogenetic analysis of OsFucT with other glycosyltrasnferase proteins. (**E**) Genotyping of homozygote (HM) and heterozygote (HT) plants to confirm the T-DNA insert. The upper lane PCR products were amplified using gene-specific primers P1 and P3 (1.2 kb), whereas the lower lane PCR products were amplified using the gene-specific primer P3 and T-DNA border sequence primer P4 (0.6 kb). (**F**) Expression analysis of *Osfuct* in Dongjin (DJ), HM and HT lines was done using RT-PCR. The expression level of *actin1* was used as a loading control and gene specific primers (P5 and P6) were used for RT-PCR analysis.

A T-DNA insertion line of mutant seeds was received from Kyung-Hee University, Republic of Korea [16]. The mutant seeds were grown under field conditions in 2012 (Suwon, Korea), and a phenotypic analysis was performed on this mutant line. The mutant displayed pleiotropic phenotypes, such as shorter height, fewer tillers and diminished shoot growth during all stages of development from seedling to maturity compared with the wild type (Figure S1). Multiple database searches including those in the Rice Annotation Project Database (RAP-DB) (Available online: http://rapdb.dna.affrc.go.jp/), Basic Local Alignment Search Tool (BLAST) (URL) of the National Center for Biotechnology Information (NCBI; available online: https://blast.ncbi.nlm.nih.gov/Blast.cgi) and the Rice Functional Genomic Express Database (RiceGE) (Available online: http://signal.salk.edu/cgi-bin/RiceGE) of the Salk Institute Genomic Analysis Laboratory were performed using the rice *Osfuct* genomic DNA sequence as a query, and these analyses revealed a single copy of the gene present at the Os08g36840 locus in the rice genome. We have examined T-DNA insertion line based on screening for distorted segregation ratios by hygromycin selection. The mutant phenotype displayed a 3:1 ratio indicating that this mutant contained a single copy of the T-DNA. The predicted mRNA was 1542 bp in length and encoded α1,3-fucosyltransferase consisting of 513 amino acids (Figure 1B,C).

Multiple sequence alignment of OsFucT with other plant glycosyltransferase proteins was performed using the ClustalW program (Available online: www.clustal.org/omega/), which revealed that α1,3-fucosyltransferase shared a conserved glycosyltrasnferase domain (Figure S2). Five transmembrane prediction programs (MEMSAT, SOSUI, TMHMM, TSEG, DAS, and HMMTOP) were used to examine the possible transmembrane region of α1,3-fucosyltransferase. A single transmembrane domain was identified in α1,3-fucosyltransferase (Figure 1C and Figure S2); the length of the transmembrane segments is underlined (Figure S2). Hence, OsFucT contains a single transmembrane domain and a conserved glycosyltransferase motif. A phylogenetic analysis was carried out using MEGA6.0 (Available online: https://www.megasoftware.net/) [17] software and the neighbor joining method. The OsFucT-encoded protein shared 83% homology with those of *Triticum aestivum* and *Hordeum vulgare* (Figure 1D). Therefore, these results suggest that *Osfuct* may have been generated from a common ancestor and the glycosyltransferase domain may have evolutionary and functional importance in plants. The mutant population was screened to identify the homozygous and heterozygous lines by polymerase chain reaction (PCR) analyses using gene-specific (P1, P2, and P3) and T-DNA border sequence primers (P4) (Figure 1A,E). We identified a mutant allele with a T-DNA insertion in the *Osfuct* gene of rice. The T-DNA was added to the first intron of the gene in the mutant (Figure 1A). The amplified PCR products were cloned and sequenced. No endogenous *Osfuct* transcript was detected in the homozygous mutant; however, the endogenous *Osfuct* transcript was observed in heterozygotes by reverse transcription-PCR (RT-PCR) analysis (Figure 1F). Thus, these results suggest that T-DNA was added in the first intron of the gene and disrupted the gene open reading frame (ORF); therefore; the *Osfuct* transcript was absent in the homozygous mutant.

### 2.2. Osfuct Mutant Significantly Affects Anther Development 

**Figure 2:**
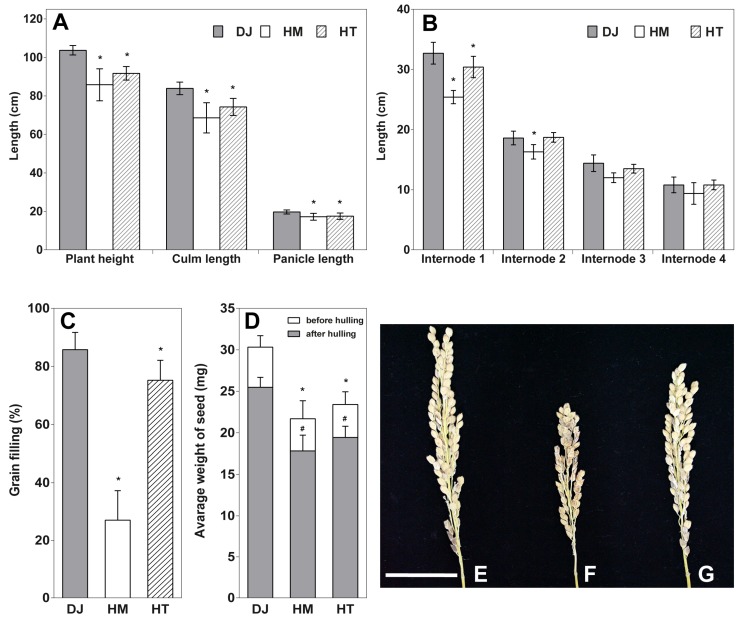
Morphological and reproductive phenotype of the *Osfuct* HM mutant and HT plant compared to Dongjin (DJ) under field conditions. (**A**) Comparison of plant height, culm length and panicle length among the DJ, HM and HT lines (*n* = 4). (**B**) Comparison of internode lengths among the DJ, HM and HT lines (*n* = 4). (**C**) Comparison of grain filling among the DJ, HM and HT lines (*n* = 4). (**D**) Comparison of average weight of 30 seeds among the DJ, HM and HT lines (*n* = 4). (**E**) Panicle phenotype of DJ. (**F**) HM. (**G**) HT lines. Scale bar is 4 cm. Data are mean ± standard deviation (SD). Asterisk indicates significant differences compared with wild type (* *p* < 0.05 by Student’s *t*-test).

**Figure 3:**
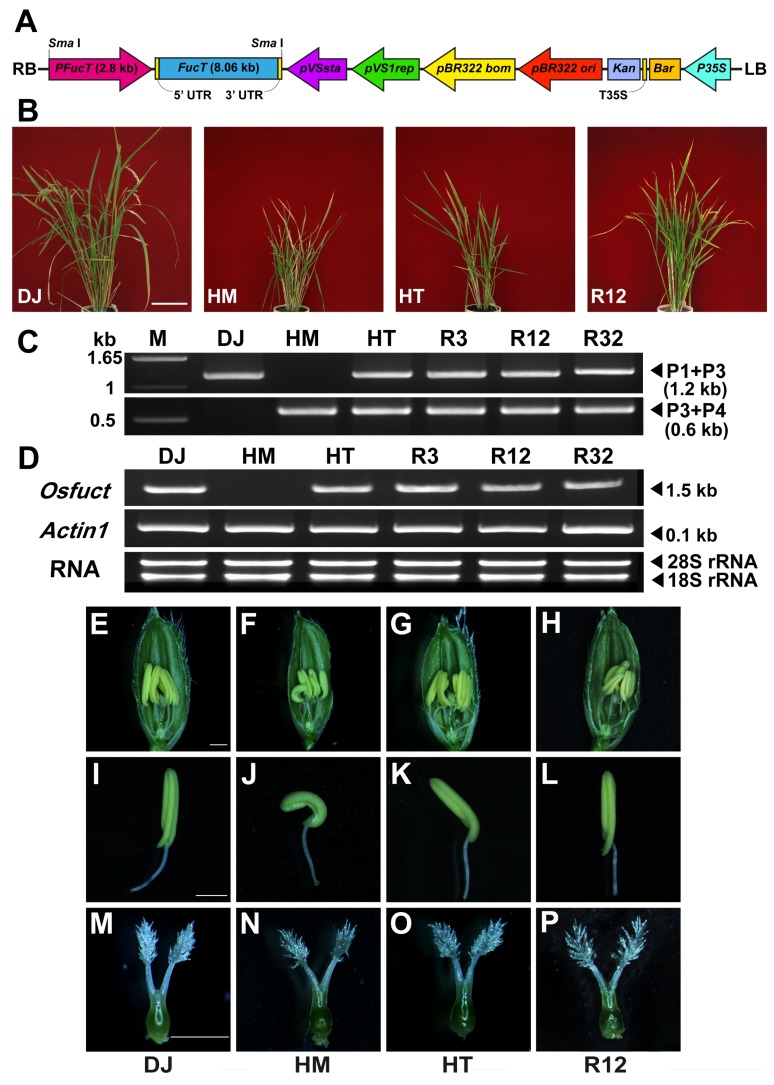
Generation of transgenic rice plants by over-expressing entire *Osfuct* gene in the HM mutant to confirm complementation. (**A**) Diagrammatic representation of the pCAMBIA3300 construct used for *Osfuct* transformation. RB, right border; FucT, entire *Osfuct* gene including the 2852-bp upstream sequence, and the 8066-bp downstream sequence; pVSsta and pVS1rep, neomycin phosphotransferase gene; pBR322 *bom* and *ori*; Kan, kanamycin resistance gene; Bar, phosphinothricin resistance gene; LB T35S, terminator of the 35S gene; *_P_FucT*, *Osfuct* promoter, LB, left border sequence. (**B**) Morphological phenotype of restored lines (R3, R12, and R32) compared with the DJ, HM and HT lines. Scale bar is 17 cm. (**C**) Genotyping of restored lines (R3, R12, and R32) compared with the DJ, HM and HT lines. Gene specific (P1, P2, and P3) and T-DNA border sequence primer (P4) used for genotyping. The upper lane PCR products were amplified using gene-specific primers P1 and P3 (1.2 kb), while the lower lane PCR products were amplified using the P3 gene-specific primer and T-DNA border sequence primer P4 (0.6 kb). (**D**) Expression analysis of the *Osfuct* transcript in the rescued lines (R3, R12, and R32) compared with the DJ, HM and HT lines using RT–PCR. The expression level of *actin1* was used as a loading control and gene specific primers (P5 and P6) were used for the RT–PCR analysis. (**E**–**L**) Comparison of anther phenotypes among the DJ, HM, HT and restored lines (R12), respectively. (**M**–**P**) Comparison of pistil phenotypes among the DJ, HM, HT and restored lines (R12), respectively. Scale bar is 1 mm.

The *Osfuct* mutant exhibited a 30% reduction in plant height and fewer tillers compared to wild-type plants; however, the heterozygous plant showed a mild phenotype (Figure 2A). The *Osfuct* mutant had shorter panicles and reduced culm lengths than those of the wild type (Figure 2A,E–G). Furthermore, elongation of all internodes in the mutant was significantly inhibited compared with the wild type (Figure 2B). Grain-filling was drastically reduced in the mutant and slightly affected in HT compared to Dongjin (Figure 2C). Seeds were smaller in the mutant compared to Dongjin (Figure 2D). Thus, these results demonstrate that α1,3-fucose transferase plays a critical role in vegetative growth and reproductive development in rice. Moreover, the mutant had a flower with a smaller panicle due to a defect in anther and pollen development, indicating that the *Osfuct* gene may have a flower development function in rice. To identify whether flower, pistil, anther, and pollen development are affected in the mutant, we examined the floral organs of mutant plants. Flowers of the wild type and mutant exhibited normal morphologies and development, including male and female organs; however, the mutant had smaller curved anthers (Figure 3F,J) compared to the wild type (Figure 3E,I). Heterozygous plants displayed less curved anthers (Figure 3G,K). The length and width of mutant flowers, anthers and pistils were significantly decreased compared to heterozygous, rescue and wild type. The average mature flower lengths of homozygous, heterozygous, rescue, and wild-type plants were 6.12 ± 0.20, 6.29 ± 0.32, 6.45 ± 0.29, and 6.78 ± 0.28, respectively (Table 1). The average mature flower widths of homozygous, heterozygous, rescued, and wild-type plants were 2.61 ± 0.25, 2.66 ± 0.34, 2.62 ± 0.22, and 2.65 ± 0.22, respectively (Table 1). The average anther lengths of homozygous, heterozygous, rescued, and wild-type plants were 1.35 ± 0.27, 1.85 ± 0.11, 1.62 ± 0.10, and 2.03 ± 0.14, respectively (Table 1). The average anther widths of homozygous, heterozygous, rescued, and wild-type plants were 0.35 ± 0.03, 0.39 ± 0.04, 0.42 ± 0.07, and 0.38 ± 0.03, respectively (Table 1). The average pistil lengths of homozygous, heterozygous, rescue and wild-type plants were 1.47 ± 0.22, 1.79 ± 0.20, 1.62 ± 0.06, and 1.74 ± 0.15 (Table 1, Figure 3M–P). The average ovule widths of homozygous, heterozygous, rescued and wild-type plants were 0.39 ± 0.02, 0.40 ± 0.03, 0.42 ± 0.01, and 0.38 ± 0.02 (Table 1, Figure 3M–P). Therefore, these results indicate that α1,3-fucose transferase plays an essential role in rice anther development.

### 2.3. Rescue of Mutant Phenotype by Wild-Type Osfuct Transgene

The mutation in the intron region of the *Osfuct* gene disrupted the ORF, which may impair the biological function of α1,3-fucosyltransferase. To assign a link between the T-DNA insertion and the mutant phenotype, we further confirmed this by a genetic complementation analysis using a genomic rescue construct. An 8066 kb genomic DNA fragment containing the entire *Osfuct* gene plus its 2852-bp promoter sequence was cloned into pCAMBIA3300 and transformed in the homozygous mutant (Figure 3A). Transgenic rice plants were selected based on their resistance to L-phosphinothricin. The mutant and α1,3-fucose-deficient phenotypes were rescued in transgenic rice plants (Figure 3B). Genotyping was done to confirm transgenic lines and further verified for the *Osfuct* transcript by RT-PCR analysis (Figure 3C,D). The rescued lines also exhibited normal morphology and development of reproductive organs and recovered their anther morphology (Figure 3H,L,P). These results show that the mutation in the intron region of the *Osfuct* gene is responsible for the pleiotropic phenotypes and demonstrate that the α1,3-fucose residue was added by *Osfuct* to the mutant (Figure S3).

### 2.4. Effect of Osfuct Mutation on Pollen Development 

**Figure 4:**
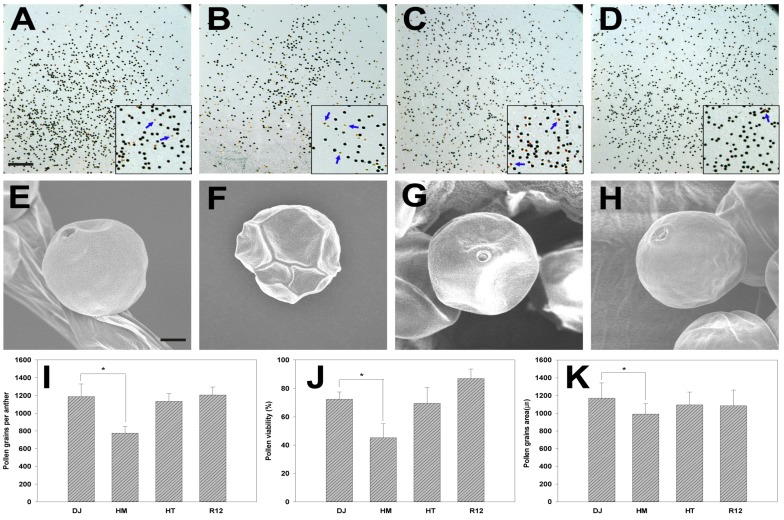
*Osfuct* mutant affects pollen morphology, viability, and total number in the mutant (HM) and HT plant compared to Dongjin. Pollen grains were stained with 80% (*w*/*v*) potassium iodide and 10% iodine. The black stain indicates viable pollen grains, whereas non-viable pollen grains are stained yellow (**A**–**D**). In inset (black box), solid blue arrow indicates the non-viable pollen (**A**) Dongjin, (**B**) HM, (**C**) HT, and (**D**) rescue (R12). Scale bar is 10 µm. Pollen morphology of mutant, rescue, and wild-type plants (**E**–**H**): (**E**) Dongjin, (**F**) HM, (**G**) HT, and (**H**) rescue (R12). The mutant produced dramatically decreased pollen grains per anther. Pollen viability test showed a reduction in total pollen number and viability in the homozygous lines compared with the wild-type, reflecting the ill-developed anther. (**I**–**K**) Measurement of pollen area (µm) in mutant (HM) and HT plant, rescue (R12), and wild type (DJ) by scanning electron microscopy (SEM). Error bars show standard deviation. Scale bar is 10 µm. Data are mean ± SD (*n* ≥ 6). Asterisk indicates significant differences compared with wild type (* *p* < 0.05 by Student’s *t*-test).

To examine anther and pollen development in flowers, anther and pollen grains were collected from HM, HT, rescued, and wild-type plants. The pollen grains were stained with 80% (*w*/*v*) potassium iodide and 10% iodine. The wild type had more viable pollen grains that stained black indicating viable pollen (Figure 4A,J). In contrast, the mutant had fewer viable pollen grains, and non-viable pollen grain stained orange (Figure 4B,J). Furthermore, to investigate the defect in pollen grains, the ultra-surface structure of the pollen grains was examined by scanning electron microscopy (SEM) in HM, HT, rescued, and wild-type plants. The pollen grains were round and well developed in both the rescued and wild-type plants (Figure 4E,H). However, the pollen grains were somewhat shriveled and significantly smaller in size in the HM line, as shown by the area of the pollen grain (Figure 4F,K). We also counted the number of pollen grains per anther; the number was significantly lower in the HM line compared with rescued and wild-type plants (Figure 4I). These results demonstrate that the mutation affects pollen morphology, viability, and pollen number per anther in rice.

### 2.5. The Glycosylation Pattern Is Altered in the Osfuct Mutant

We performed multiple sequence alignment of α1,3-fucosyltransferase amino acid sequences with homologs from other plant species. The OsFucT homologous protein sequences were retrieved from the National Center for Biotechnology Information database (Available online: http://www.ncbi.nlm.nih.gov/). The alignments revealed that α1,3-fucosyltransferase shared a highly conserved glycol transferase domain. α1,3-Fucosyltransferases are responsible for transferring the α1,3-linked fucose residue to the glycoprotein *N*-glycan. To investigate the glycosylation pattern, HM, HT, rescued and wild-type plants were subjected to total *N*-glycan analysis by mass spectrometry. Lack of fucose residues in the *N*-glycan mutant was examined by subtracting 146 mass units from rescued and wild-type plants. The mass spectra derived from the mutant were significantly different from those of the wild-type and rescued lines (Figure S3A–D). The fucose residue was missing from the *N*-glycan structure of the mutant. The four major peaks of the complex-type *N*-glycans differed in the mutant and wild type (Figure S3E, Table S3): Man_3_XylFucGlcNAc_2_ (*m*/*z* 1211.3), GlcNAcMan_3_XylFucGlcNAc_2_ (*m*/*z* 1414.6), GlcNAc_2_Man_3_XylFucGlcNAc_2_ (*m*/*z* 1617.4), and (FA) (FA)XF_3_ (*m*/*z* 2235.6). These results suggest that inactivation of the *Osfuct* gene in the mutant produced *N*-glycans without α1,3-fucose residues, whereas the rescued lines were capable of producing the complex *N*-glycans including the α1,3-fucose residue.

### 2.6. Osfuct Mutant Exhibits Sensitivity to Salt Stress

**Figure 5:**
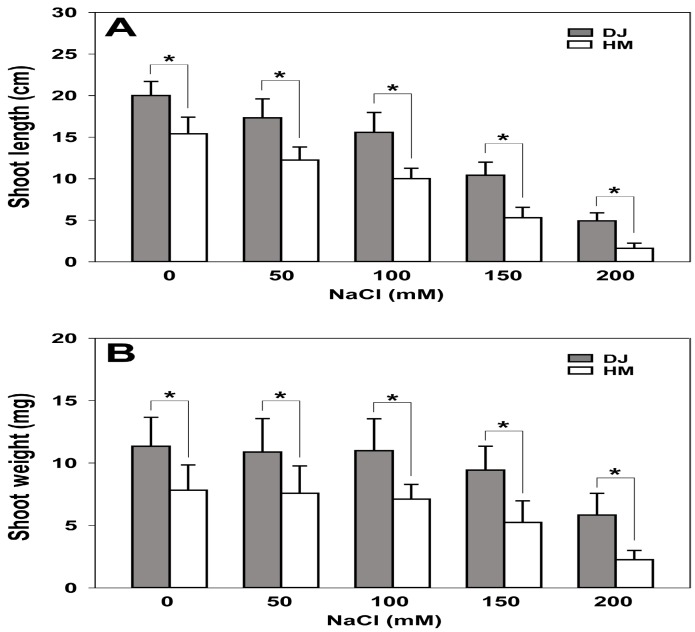
*Osfuct* mutant sensitivity to salt stress. Effect of salt stress on growth of rice seedlings. Seedlings of the Dongjin (DJ) and mutant lines (HM) were exposed to a nutrient solution containing 0–200 mM NaCl. After 3 weeks, individual leaves were separated and shoot length (**A**) and weight were measured (**B**). Data are mean ± SD (*n* = 15). Asterisk indicates a significant difference compared with wild type (* *p* < 0.05 by *t*-test). Error bars show standard deviation.

We performed shoot growth assays to investigate salt tolerance in the mutant on MS medium supplemented with 0, 50, 100, 150, and 200 mM NaCl (Figure 5A,B). However, the mutant plants were smaller compared to the wild type. Growth was monitored for 2 weeks, and the mutant exhibited sensitivity to salt stress and overall growth in comparison to the wild type. The mutant plants exhibited pale-green leaves phenotype compared to the wild type. The plants were most sensitive to the highest concentration of NaCl (200 mM). The most sensitive phenotype was evaluated in term of shoot growth over 14 days, and all mutant lines showed sensitivity and poor growth at the highest concentration of NaCl (200 mM). The fresh weight of wild-type shoots was measured after 2 weeks of growth at 0, 50, 100, 150, and 200 mM. The values were: 11.34 ± 2.32, 10.88 ± 2.67, 10.98 ± 2.55, 9.42 ± 1.93, and 5.83 ± 1.72 mg, whereas mutant shoots weighed only 7.81 ± 2.02, 7.57 ± 2.20, 7.09 ± 1.19, 5.23 ± 1.72, and 2.25 ± 0.74 mg, respectively (Figure 5A,B). In addition, a positive correlation was found between increase salt concentration and proportionally reduction of fresh weight of mutant plants. These results suggest that this rice mutant is sensitive to high salt concentrations.

### 2.7. Transcriptomic Profiling of the Osfuct Mutant

We performed a whole transcriptome analysis of the mutant (HM) and wild type (Dongjin) to identify the possible underlying molecular mechanisms that could explain the pleiotropic functions of *Osfuct* during growth and reproductive development. To investigate the effect of *Osfuct* mutation on transcriptional regulation of other pathway genes, whole transcriptomic profiling was performed with three biological replicates in mutant and wild type (Dongjin) plants, respectively. The rice whole genome 135 K oligo microarray (GreenGene Biotech., Inc., Seoul, Korea) was used for the transcriptome profiling (Figure S4). The transcriptome analysis and data normalization were carried out as described in the Materials and Methods section (Figures S5 and S6, Tables S6 and S7). Differentially expressed (either up or downregulated) transcripts between the mutant and wild type were determined via log_2_ fold change above 2.0 (*p* < 0.05) and log_2_ fold change above 1.6 (*p* < 0.1) change thresholds. Among the 42 significantly differentially expressed genes, 32 were upregulated (Table 2 and Table S4), and 10 were downregulated (Table 2 and Table S5). We categorized those genes into two groups to make the broad knowledge and interpret our microarray data clearly. Group 1 genes included those with the log_2_ fold change above 2 expression change threshold (*p* < 0.05), while group 2 genes included those with the log_2_ fold change above 1.6 threshold (*p* < 0.1). The expression of several key genes involved in plant growth and development was altered in the mutant compared with the wild type (Table 2, Tables S4 and S5). Protein kinases, transcriptional factors, metabolism-related genes, and hypothetical genes comprised a significant portion of the differentially expressed genes in the mutant compared with the wild type. The following genes were significantly upregulated: putative disease resistance protein RGA4 required to recognize *Avr* (AVR1-CO39) to mediate disease resistance [18]; LRR (Leucine-rich repeat)receptor-like serine/threonine-protein kinase regulating the diverse developmental processes [19]; putative wall-associated protein kinase involved in cell expansion and response to external stimuli [20]; F-box protein-like implicated in phytohormone signaling [21]; isocitrate lyase involved in glyoxylate pathway [22]; disheveled-associated activator of morphogenesis 1 involved in regulating the actin cytoskeleton [23]; glutathione S-transferase implicated in abiotic tolerance [24]; transcription factor (basic helix-loop-helix) bHLH35 regulating diverse biological processes including epidermal differentiation, seed germination, carpel and anther development, response to phytochrome, diverse biotic and abiotic stresses [25]; Dof zinc finger protein involved in wide range of fundamental processes in higher plants [26]; transcription initiation factor IIE involved in gene expression [27]; eukaryotic translation initiation factor 4A-3 required for protein synthesis [28]; and hypothetical proteins. Significantly downregulated genes included glycoprotein 3-α-l-fucosyltransferase A function in *N*-glycosylation [1,2]; the WASH complex subunit CCDC53 homolog regulating actin polymerization [29]; kinesin-like protein (KIF19) and cortical cell-delineating protein involved in morphogenesis, mitosis, and signal transduction [30]; endosperm defective 1 (EDD1) required for microtubule function [31]; glycine-rich RNA-binding protein 2 play crucial roles in post-transcriptional gene regulation [32]; l-type lectin-domain containing receptor kinase in plant developmental processes including signal transduction, response to hormone, biotic and abiotic stresses [33]; non-specific lipid transfer protein implicated in key cellular processes including cell wall organization, the stabilization of membranes, and signal transduction [34]; and some hypothetical genes.

**Figure 6:**
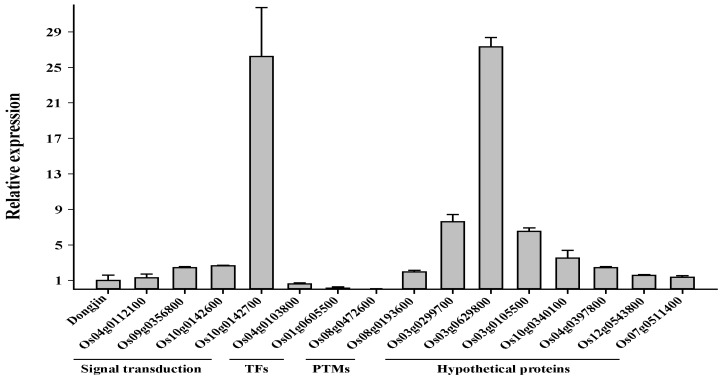
Quantitative RT-PCR (qRT-PCR) results for the differentially expressed transcripts in wild type (DJ) and the *Osfuct* mutant (HM) to verify transcriptome profile data produced by microarray analysis. Data are mean ± SD (*n* = 3).

**Figure 7:**
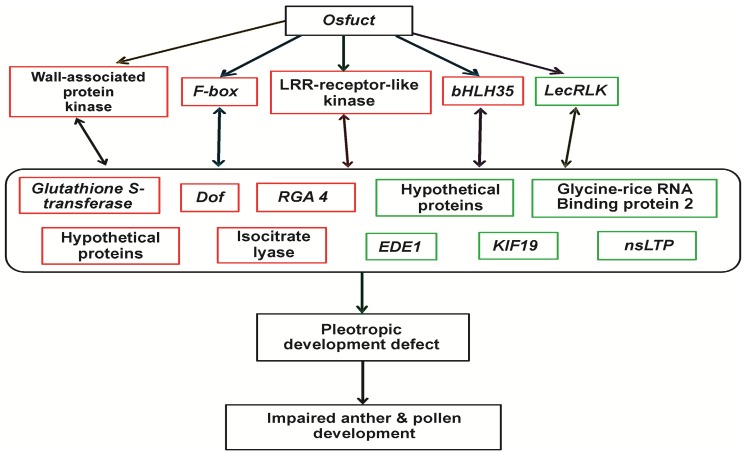
Possible function of *Osfuct* gene in growth, anther, and pollen development in rice. All up and downregulated genes together may affect growth and development in rice. Upregulated genes are in the red box and downregulated genes are in the green box. LecRLK, lectin receptor-like kinase; LRR-RLK, leucine-rich repeat receptor-like kinase; bHLH, basic/helix-loop-helix; DOF, DNA-binding one zinc finger; nsLTPs, non-specific lipid-transfer proteins; RGA4, resistance; EDE1, ENDOSPERM DEFECTIVE 1; KIF19, kinesin-like protein 19.

To further verify whether the transcript changes identified by the microarray analysis were reliable, total RNA from the same mutant and wild type was subjected to quantitative RT-PCR (qRT-PCR) analyses. The 42 genes that were either significantly up- or down-regulated in the microarray analysis were selected for qRT-PCR analysis (Table 2, Tables S4 and S5). We observed a significant correlation between the qRT-PCR and microarray results. Although some differences were found between the microarray and qRT-PCR analysis, the pattern of differentially expressed genes detected by the two approaches remained consistent. Thirty-two (72%) of the 42 genes corresponded to the microarray results, and the expression of 10 genes varied in both approaches (Figure 6 and Figure S7). Based on the earlier reported functions of these up- and downregulated genes [18,19,20,21,22,23,24,25,26,27,28,29,30,31,32,33,34] we proposed a possible working model in Figure 7 to depict the underlying molecular mechanism of *Osfuct* function in the pleiotropic development defects in rice. This model demonstrates that loss of function *Osfuct* modulates the genes involved in plant developmental processes, such as transcriptional factors and protein kinases, which affect growth as well as anther and pollen development in later stages.

## 3. Discussion

In this study, we demonstrated that loss of *Osfuct* dramatically impeded growth and flower development in rice. To our knowledge, this is the first report on the function of *Osfuct* in anther and pollen development in the *Osfuct* mutant. In addition, the anther was curved in the HM mutant (Figure 3F,J), while the anther was less curved and showed a milder phenotype including plant height, tiller number, panicle lengths and elongation in the HT plant (Figure 3G,K) compared with the wild type (Figure 3E,I). A single copy of the functional *Osfuct* allele may compensate for partial function in the HT plant. We provide strong evidence that loss of *Osfuct* impairs pollen and anther development in the mutant. 

Microarray analyses revealed that several genes essential in plant developmental processes were significantly altered in *Osfuct* mutant compared to wild-type. Receptor-like protein KINASE2 (RPK2) T-DNA insertional mutants exhibit defects in anther and pollen development in *Arabidopsis* [35]. LRR-RLK and MSP1 (Multiple Sporocyte1) determine anther cell identity in rice [36]. Similarly, downregulation of Embryonic flower 2b (OsEMF2b) causes defects in anther and pollen development in rice [37]. Our microarray results show that the expression of leucine-rich repeat receptor-like kinase (LRR-RLK) was significantly elevated in the mutant (Table 2). bHLH transcription factor called Dysfunctional Tapetum1 (DYT1) is essential for anther development in *Arabidopsis* [38]; the transcript level of bHLH transcription factor was also significantly up-regulated in the mutant. There are several studies showing that (gibberellic acid) GA synthesis and signaling are important for anther and pollen development. Arabidopsis loss-of-function mutant in copalyl diphosphate synthase (*ga1*) has poorly developed anthers and pollen grains also are not viable [39]. Our results are agreement with the previous findings and suggest that *Osfuct* may affect anther and pollen development processes by altering the expression of genes implicated in gametophyte development in rice. However, how *Osfuct* participates in pollen and anther development remains elusive (Figure 3E–L and Figure 4A–K). 

The *Osfuct* mutant displayed pleiotropic developmental defects, such as shorter plant height, fewer tillers, shorter panicle length and internode elongation (Figure 2A,B and Figure 3B). We recorded all phenotypic data from the field-grown plants to avoid the conditional phenotypic variation. MYB-like gene of plant height 1 (*OsMPH1*) has been implicated in the regulation of plant height through change internode cell length in rice. Overexpression of *OsMPH1* leads to increases of plant height and grain yield, while knockdown of *OsMPH1* showed the opposite phenotypes in rice. RNA-sequencing data also revealed that expression profile of cell elongation and cell wall synthesis-related genes significantly modulated in transgenic rice plants [40]. Dwarf Tiller1 (DWT1) regulates the shoot and tiller formation in rice. Mutant plants had main shoots with normal height and larger panicles but dwarf tillers had smaller panicles compared to the wild-type. The *DWT1* gene is highly expressed in young panicles. Mutant was defective in internode elongation is associated with gibberellin signaling [41]. Rice ethylene-response AP2/ERF (*OsEATB*) factor regulate the internode elongation by repressing a gibberellin biosynthetic gene, ent-kaurene synthase A [42]. Our results also consistent with these findings and indicate that *Osfuct* may possess important biological and developmental functions in rice. The expression level of DNA-binding one zinc finger (DOF) in the *Osfuct* mutant increased significantly as well; the DOF transcription factor family has been implicated in a wide range of fundamental processes in higher plants, including seed germination, maturation, photosynthesis, flower induction, responses to phytochrome, plant hormone signaling, and biotic and abiotic stressors [26]. Overexpression of *OsDof12* in rice reduces height, shortens leaf length, produces more erect leaves and smaller panicles, and decreases grain yield in transgenic rice plants [43]. Collectively, these results demonstrated that loss of *Osfuct* may modulate the expression of genes participating in plant growth and developmental processes.

In addition, the transcript levels of the F-box proteins increased significantly in the mutant compared to wild type through microarray data; F-box proteins have also been implicated in phytohormone signaling through ubiquitin-mediated protein degradation [44]. An optimal concentration of phytohormones is essential for various developmental processes, such as embryogenesis, tissue differentiation, and organogenesis. The *fuct-1* and *fuct-2* mutants affect basipetal auxin transport and accumulation resulting in decreased gravitropic responses in rice [15]. Our results were consistent with earlier findings that the lack of fucose residues in the mutant may affect the function of the key genes involved in phytohormone signaling and plant developmental processes, which subsequently may affect growth and development in the mutant [15]. However, how *Osfuct* regulates the underlying molecular mechanisms of plant developmental processes remains to be established. Furthermore, the pollen grains were shrunken and significantly smaller in size in the mutant compared to the restored and wild type. Additionally, pollen viability and pollen number per anther decreased dramatically in the mutant (Figure 4A–K). The mutant seeds were smaller and the total number of seeds per plant also decreased. Pollen development is also defective in gibberellin-deficient mutants *gib-1* and *gib-2* in the tomato [45]. Lalanne et al. [9] demonstrated that a defect in *SETH1* and *SETH2* affects pollen germination, tube growth, and male transmission in *Arabidopsis*. *SETH1* and *SETH2* are involved in the first step of the glycosylphosphatidylinositol biosynthetic pathway. Mutation in two phytochrome genes (*PHYA* and *PHYB*) affect the anther and pollen development in rice [46]. Isocitrate lyase is a key enzyme in the glyoxylate pathway that is widespread in nature and functions in diverse physiological processes in many organisms [22]. Isocitrate lyase and malate synthase genes from *Brassica napus* are active at specific stages of pollen development [22]. Our microarray analyses demonstrated that the isocitrate lyase transcript level was significantly induced in the mutant. Loss of *Osfuct* may affect expression of these genes and also deregulate genes that play an essential role in anther and pollen development. Further, the transcript level of kinesin-like protein KIF19 decreased significantly as wellin the mutant. AtNACK1 and AtNACK2 are members of the kinesin-7 family essential for cell-plate formation in pollen and male and female gametogenesis [47]. Similarly, the pollen semi-sterility 1 (*PSS1*) mutant encodes a kinesin-1-like protein affecting spikelet fertility with reduced pollen viability and defective anther dehiscence in rice [48]. These results indicate that loss of *Osfuct* may result in aberrant regulation of the expression of genes implicated in anther and pollen development. However, regulation of pollen development processes by *Osfuct* remains unclear. 

To verify the mutant phenotype, we conducted a genetic complementation test and introduced the entire *Osfuct* gene, including the upstream region, into the mutant. Genetic complementation analyses revealed that the transgenic rice lines restored the pleiotropic phenotype of mutant (Table 1; Figure 3B and Figure 4D,H–K); hence, the mutation in the intron region of *Osfuct* was responsible for the diverse developmental abnormalities. Two rice mutants (*fuct-1* and *fuct-2*) with loss of *Osfuct* function displayed larger tiller angles, shorter panicle lengths and internodes, reduced grain filling, and an increase in the number of chappy grains with unusual shapes [15]. They also performed a complementation analysis and mobilized the cDNA with a promoter containing a portion of the first exon and restored the phenotype. Taken together, these results suggest that *Osfuct* may have pleiotropic functions in various developmental processes in rice. 

*N*-glycosylation plays an important role in regulating protein folding, subcellular localization, and function of secreted proteins. Mutants interrupted in *N*-glycosylation biosynthesis have impaired reproduction and embryo formation [50]. OsDGL1 homologs of the oligosaccharyltransferase complex subunit have been implicated in *N*-glycosylation and mutation in *Osdgl1*, causing a defect in *N*-glycosylation in rice. The *Osdgl1* mutant modulates root cell wall polysaccharide composition, leads to a smaller root meristem, shorter root cells, and root cell death [8]. α1,3-Fucosyltransferase and β1,2-xylosyltransferase are deactivated by multiplex CRISPR/Cas9 in *Nicotiana tabacum* BY-2 cells that produce glycoproteins deficient in plant-specific *N*-glycans [14]. Our results also demonstrate that the mutant exhibited pleiotropic phenotypic defects, including impaired growth, fewer tillers, shorter plant height, panicle, culm, impeded internode elongation, and defects in anther and pollen development under field conditions (Figure 2). These results indicate that missing fucose residues in the *N*-glycan complex in the mutant affected the expression of genes that play a crucial role in plant developmental processes. How *N*-glycosylation affects vegetative and reproductive development remains to be determined. Moreover, the *Osfuct* mutant was sensitive and showed poor growth at increased salt concentration. As a previous study showed, that defect in *N*-glycan synthesis, processing, and maturation of complex glycan 1 (*cgl1*) makes the plants more salt-sensitive compared with the wild type [11]. Thus, the proper maturation of *N*-glycan in the Golgi complex is essential for salt tolerance in *Arabidopsis* [14]. *Arabidopsis* T-DNA insertion mutant (*alg10-1*) encoding α1,2-glucosyltransferase exhibits a severe defect in *N*-glycosylation, leaf growth, and increased salt sensitivity [13]. Transcriptome results also show that the glutathione S-transferase expression was upregulated more than threefold in the mutant. Glutathione S-transferases are crucial detoxification enzymes involved in abiotic stress tolerance by metabolizing numerous toxic compounds through glutathione conjugation and play a critical role improving tolerance to cold, osmotic dehydration, salt, and herbicide-induced damage [24]. Ectopic expression of glutathione S-transferase enhances salt and drought tolerance in *Arabidopsis* [51]. The transcript level of lectin receptor-like kinase (LecRLK) decreased significantly in the mutant. LecRLK is known to play an important role in plant developmental processes, including signal transduction and responses to hormone, biotic, and abiotic stressors [33]. The expression of non-specific lipid-transfer proteins (nsLTPs)_was significantly repressed in the *Osfuct* mutant. nsLTPs are also play an essential role in plant growth and development, sexual reproduction, seed germination and development, tolerance to biotic and abiotic stress [34]. Our results also show that the *Osfuct* mutant was sensitive to the highest concentration of NaCl (200 mM) and that the fresh weight of shoots was proportionally reduced compared to wild-type plants (Figure 5A,B). Therefore, it is intriguing to assume that a deficiency of α1,3-fucose may affect key signaling pathways and the expression of genes related to the abiotic stress response in rice. However, it is unknown how OsFucT regulates the abiotic stress response in the mutant via *N*-glycosylation. Finally, expression of 19 hypothetical genes was dramatically induced/repressed (induced 17; repressed 2) in the mutant; thus, it is impossible to speculate about their possible effects on rice growth and development at present. 

In summary, we characterized a novel *Osfuct* mutation that significantly affects anther and pollen development in rice. The loss of *Osfuct* caused pleiotropic morphological abnormalities, such as shorter plants, reduced panicle length, inhibited internode elongation, and impaired anther and pollen development. Several genes involved in growth and development processes were also drastically altered in the mutant. Thus, collectively our results demonstrate that *Osfuct* plays an essential role in anther and pollen development and has potential applications in a rice-breeding program for producing humanized *N*-glycan proteins. Several questions remain to be addressed. How does *Osfuct* act at the cellular level? Identification and characterization of *Osfuct*-interacting partners to dissect the intricate regulatory gene network may shed further insight into the functions of *Osfuct* impaired growth, anther, and pollen development.

## 4. Materials and Methods 

### 4.1. Plant Materials and Growth Conditions for Mutant Screening

The T-DNA insertion line of mutant seeds (PFG_2A-30078.L) was obtained from Kyung-Hee University, Republic of Korea [19]. The rice (*Oryza sativa* cv Dongjin) and mutant seeds were sterilized by dipping in 70% ethanol for 1 min and washed three times with double-distilled water. The seeds were sterilized repeatedly with 2% sodium hypochlorite for 30 min, washed five times with double-distilled water, and germinated on 2N6 media in a growth chamber under a 16-h light (28 °C)/8-h dark (26 °C) photoperiod during the full life cycle. The seedlings were transferred to individual pots and grown under field conditions during the 2012 season (Suwon, Korea) to measure the morphological phenotypes of rice. 

### 4.2. Genotyping and Phylogenic Analysis of the Osfuct Mutant

T-DNA insertion and homozygosity were verified by genomic PCR using primers specific to *Osfuct* and T-DNA. Genomic DNA was extracted from leaves of 4-week-old plants according to the CTAB protocol [52]. Genomic DNA purity and concentration were measured using a NanoDrop spectrophotometer (ND-1000; Nanodrop Technologies, Wilmington, DE, USA). T-DNA insertion and homozygosity of *Osfuct* were confirmed by the gene-specific primer pair (P1 + P3), the T-DNA left border, and the gene-specific primer (P4 + P3) used in the PCR (Biometra TProfessional ThermoCycler, Gottingen, Germany; Phire^®^ Hot Start DNA Polymerase, Finnzymes, Ipswich, MA, USA). The PCR was carried out with the initial reaction of 98 °C for 5 min; followed by 35 cycles of 98 °C for 5 s, 67 °C for 5 s, and 72 °C for 1 min; with a final reaction of 1 min at 72 °C. All primers used in the analysis are presented in Table S1.

Evolutionary history was inferred using the neighbor-joining method. The bootstrap consensus tree inferred from 1000 replicates was taken to represent the evolutionary history of the species (sequence) for analysis. The deduced amino acid sequence of α1,3-fucosyltransferase (XP_015650720) was compared with glycosyltransferase proteins from *Arabidopsis thaliana* (XP 020888577), *Triticum aestivum* (CAE46649), *Hordeum vulgare* (CAE46648), *Setaria italic* (XP_004973669), *Zea mays* (NP_001105927), *Sorghum bicolor* (XP_002444504), *Cajanus cajan* (XP_020208222), *Glycine max* (XP_003518836), *Nicotiana tabacum* (NP_001311874), *Solanum lycopersicum* (XP_004230780), *Selaginella moekkendorffii* (XP_002960463), and *Physcomitrella patens* (CAD22109).

### 4.3. Reverse Transcription-Polymerase Chain Reaction (RT-PCR) and Quantitative Real-Time PCR (qPCR) Analysis

Total RNA from frozen rice leaves was extracted in liquid nitrogen [53]. Briefly, frozen rice leaves were ground to powder and transferred to a microcentrifuge tube. A total of 800 µL of extraction buffer (200 mM Tris-Cl, 400 mM LiCl, 25 mM EDTA, and 1% SDS, pH 9.0) and 600 µL of acidic phenol was added, vortexed for 10 s, and the solution was kept on ice for 30 min followed by centrifugation at 10,000× *g* for 2 min at 4 °C. The supernatant was transferred to a microcentrifuge tube and 600 µL phenol and chloroform were added and centrifuged at 10,000× *g* for 5 min at 4 °C. The supernatant was transferred to a new microcentrifuge tube and precipitated in 250 µL of 8 M LiCl at −70 °C overnight. After centrifugation, the pellets were washed twice with 70% cold ethanol, completely dried, and dissolved in (Diethyl pyrocarbonate) DEPC-treated distilled water. Genomic DNA contamination was removed by treating with DNase I (Takara Bio. Inc., Shiga, Japan) at 37 °C for 30 min and cDNA was generated by reverse transcribing the mRNA using the iScript^TM^ cDNA synthesis kit (Bio-Rad, Hercules, CA, USA) at 46 °C for 20 min. cDNA was quantified using a spectrophotometer (ND-1000), and subjected to RT-PCR analysis using the primer set (P5 + P6) with the following PCR profile: 40 cycles of 98 °C for 10 s, 55 °C for 5 s, and 72 °C for 1 min. The *actin1* (XM_015774830) primer was used as an internal loading control. Amplified RT-PCR products were further confirmed by DNA nucleotide sequencing. Quantitative real-time PCR (qPCR) was performed using a CFX96 Real-Time PCR Detection System (Bio-Rad) with the SYBR Premix (Toyobo, Osaka, Japan). The qPCR reactions were run with the following profile: denaturation at 95 °C for 1 min, followed by 39 cycles of denaturation at 95 °C for 15 s, and annealing at 55–60 °C for 30 s. A melting curve was obtained through a protocol of 95 °C for 1 min, 95 °C for 15 s, 65 °C for 1 min followed by cooling at 40 °C for 10 min. qPCR was performed with three technical replicates and repeated three times for each gene. CFX Manager 2.0 (Bio-Rad) was used to calculate the cycle threshold value. Rice ubiquitin1 (*OsUBQ1*, XM_015774309) was used as the control to normalize the data. Primer pairs were designed using PrimerQuest Tool (Integrated DNA Technologies, Coralville, IA, USA) and all primers used in this study are listed in Table S2.

### 4.4. Phenotypic Analyses of the Osfuct Mutant

Seeds of *Osfuct* and wild-type rice were sown and transferred to pots to grow under field conditions following the full lifecycle for a phenotypic examination during different developmental stages. To evaluate morphological phenotypes including plant height, tiller number, shoot, culm, panicle, and internode elongation length, the mutant and wild-type (Dongjin) rice were grown under field conditions during the 2012 season (Suwon, Korea) to follow the full lifecycle. The mutant and wild-type (Dongjin) rice were grown in greenhouse conditions to analyze reproductive development parameters, such as length and width of mature flowers, anthers, pistils, pollen grain viability, and pollen grains per anther. 

### 4.5. Assessment of Reproductive Development of the Osfuct Mutant

Flowers were collected from mutant and wild-type rice plants to analyze reproductive development. The anther and pistil images were taken with a microscope (SteREO Discovery V12, Carl Zeiss, Zena, Germany). To examine the ultrastructure of pollen, *Osfuct* (HM, homozygote, HT, heterozygote), rescued, and wild-type pollen images were captured by a JEOL JSM–7500FA (JEOL Ltd., Tokyo, Japan) analytical field-emission scanning electron microscope after coating with tungsten particles using ion (magnetron) sputter coater (Hitachi E-1030) in vacuum. The pollen grains were stained with 80% (*w*/*v*) potassium iodide and 10% iodine to determine pollen viability. 

### 4.6. Vector Construction for Complementation Analysis of Osfuct Disrupted Mutant

An 8066-bp fragment of genomic DNA plus a 2852-bp upstream sequence that contained the promoter region cloned into the *Sma*I site of the binary vector pCAMBIA3300 after modification of restriction enzyme sites were used for the complementation analysis of the mutant. The promoter, genomic, and cDNA fragments were amplified using the primer pairs (P7 + P6) and (P5 + P6) and the PCR profile described above. Nucleotide sequences of the cloned fragments were verified by an automated DNA sequencer (3730xI DNA Analyzer, Applied Biosystems, Foster City, CA, USA). These plasmid constructs were electroporated into the *Agrobacterium tumefaciens* strain LBA4404 and introduced into the homozygote mutant by *A. tumefaciens*-mediated transformation with some modifications as described previously [54]. Transgenic rice plants were selected on l-phosphinothricin (6 mg/L) containing medium. l-phosphinothricin resistance transgenic rice plants were transferred to soil and allowed to grow in a greenhouse, as described above, for further phenotypic analysis.

### 4.7. N-Glycan Profiling of the Osfuct Mutant

*N*-glycans were prepared and purified from the mutant and wild-type rice leaves according to Karg et al. [55]. The *N*-glycan analysis was performed in positive-ion reflectron mode using a matrix-assisted laser desorption ionization-time of flight mass spectrometer (MALDI-TOF MS) with an Autoflex system from Bruker Daltonics (Bruker, Billerica, MA, USA). The tandem mass spectrometric analysis was carried out using an Axima Resonance MALDI-quadrupole ion trap-TOF instrument (Shimadzu, London, UK). 5-Dihydroxybenzoic acid (10 mg/mL in 50% methanol) was used as the matrix. The data analysis was performed with Flex Analysis 3.3 software (Bruker) and Launchpad 2.9.3 software (Kratos Analytical Ltd., Cambridge, UK). The calculated mass of the major *N*-glycans was compared with earlier reports to assign the corresponding structures [12,13,56].

### 4.8. Salt Tolerance Assay of the Osfuct Mutant

Young mutant plants were subjected to high salinity stress assay. The young mutant and wild-type rice plants were grown in 0, 50, 100, 150, and 200 mM NaCl-containing medium for 2 weeks. Shoot length and weight were scored, and the plants were photographed. Four biological replicates of rice plants were used for the salt tolerance assay. 

### 4.9. Transcriptome Analyses of the Osfuct Mutant

Seeds of mutant (homozygote; HM) and wild-type (Dongjin) rice were sterilized and germinated as previously described in Section 4.1 in the growth chamber. Seedlings were transferred to individual pots and grown in a greenhouse. Three biological replicates of HM and Dongjin rice plants were selected respectively for transcriptome analysis using the rice whole genome 135 K oligo microarray (GreenGene Biotech., Inc., Seoul, Korea). These microarrays contained 31,439 genes submitted to the International Rice Genome Sequencing Project (IRGSP, available online: http://rgp.dna.affrc.go.jp/E/IRGSP/) and the Rice Annotation Project version 2 (RAP2, available online: http://rapdb.dna.affrc.go.jp/). Total RNA was extracted from leaves of 4-week-old plants at the tillering stage, and genomic DNA was removed by DNase I (Takara Bio) digestion. Double-stranded cDNAs were synthesized using the RevertAid^TM^ H Minus First Strand cDNA Synthesis Kit (Fermentas, Vilnius, Lithuania) according to the manufacturer’s directions. Briefly, total RNA (10 µg) was mixed with following components (4 µL of 5× first strand buffer, 1 µL of oligo dT primer (100 µM), 1 µL RiboLock^TM^ Ribonuclease inhibitor, 2 µL of 10 mM deoxynucleotide mix and 1 µL of RevertAidTM H Minus M-MuLV Reverse transcriptase enzyme) and incubated at 42 °C for 1 h. Reverse transcription and (Double-stranded DNA) dsDNA synthesis were terminated by incubating at 70 °C for 10 min. Cy3-labeled DNAs were synthesized by adding the following master mix (1 µg of double-stranded cDNA, 30 µL of Cy3-9mer primers, 2 µL of Klenow fragments (50 U/µL, Takara), 10 µL of 50× deoxynucleotide mix (10 mM each), and 8 µL of double distilled water) and incubated at 37 °C for 2 h. The labeled dsDNA was purified using the MinElute Reaction Cleanup Kit (Qiagen, Valencia, CA, USA) and hybridization was carried out with the MAUI chamber (Biomicro, Salt Lake City, UT, USA) at 42 °C for 16–18 h. After hybridization, the microarrays were washed at 42 °C for 10–15 s with GE Wash Buffer I, again for 2 min at 42 °C with GE Wash buffer II, and finally washed with Wash buffer III for 1 min with agitation. The microarray was dried immediately by centrifugation at 500× *g* for 1 min and scanned using GenePiX scanner 4000 B (Molecular Devices, Inc., Sunnyvale, CA, USA). The signal was digitized and analyzed by Nimblescan (Madison, WI, USA). To increase the sensitivity and reproducibility of the transcriptome analysis, the data were normalized using the cubic spline normalization method, and probe-level summarization was processed by Robust Multichip Analysis using a median polish algorithm. Fold-change was calculated using Acuity 3.1 (Axon Instruments, Sunnyvale, CA, USA). Greater than two- or log_2_ fold change above 1.6 with *p* < 0.05 (or 0.1) was set as the threshold for statistical significance. The *Osfuct* mutant gene expression profiles have been deposited in NCBI’s Gene Expression Omnibus and are accessible through Gene Expression Omnibus (GEO) Series accession number GSE110873 (Available online: https://www.ncbi.nlm.nih.gov/geo/query/acc.cgi?acc=GSE110873).

## Figures and Tables

**Table 1:** Morphological comparison of flowers, pistil, and stamen parts of the wild type (DJ), HM, HT, and complemented line (R12).

Floral Parts	Dimensions (mm)	DJ	HM	HT	R12
Mature flower	Length (mm)	6.78 ± 0.28	6.12 ± 0.20 *	6.29 ± 0.32 *	6.45 ± 0.29
	Width (mm)	2.65 ± 0.22	2.61 ± 0.25	2.66 ± 0.34	2.62 ± 0.22
Anther	Length (mm)	2.03 ± 0.14	1.35 ± 0.27 *	1.85 ± 0.11 *	1.81 ± 0.07 *
	Width (mm)	0.38 ± 0.03	0.35 ± 0.03	0.39 ± 0.04	0.38 ± 0.01
Pistil	Length of pistil (mm)	1.74 ± 0.15	1.47 ± 0.22 *	1.79 ± 0.20	1.77 ± 0.15
	Width of the ovule (mm)	0.38 ± 0.02	0.39 ± 0.02	0.40 ± 0.03	0.38 ± 0.01

Data are mean ± standard deviation (SD). Asterisk indicates significant differences compared with wild type (* *p* < 0.05 by Student’s *t*-test). Different numbers of floral parts from DJ, HM, HT (*n* ≥ 9) and R12 (*n* ≥ 3) were used in this experiment.

**Table 2:** Microarray genes significantly (−log_2_ fold change >2 and *p* < 0.05) up- and down-regulated in the *Osfuct* mutant (HM) compared with (DJ).

SN	Gene ID	Gene Location	DJ vs. HM (log_2_ Fold Change)	Gene Descriptions
1	Os03g0299700	10495846..10498081	46.75	Hypothetical protein
2	-	-	32.96	Hygromycin *
3	Os03g0629800	24806433..24807496	24.06	Hypothetical protein
4	Os01g0965300	42554248..42554866	14.77	Hypothetical protein
5	Os03g0105500	338364..338744	8.03	Hypothetical protein
6	Os02g0269650	9689236..9689758	5.41	Hypothetical protein
7	Os10g0340100	9458993..9461016	5.14	Hypothetical protein
8	Os04g0112100	692745..695801	4.55	Putative disease resistance protein RGA4
9	Os09g0356800	11483610..11506480	4.38	LRR receptor-like serine/threonine-protein kinase
10	Os04g0397800	19645507..19646962	4.32	Hypothetical protein
11	Os12g0543800	21881810..21883709	4.29	Hypothetical protein
12	Os07g0511400	20194023..20194735	3.48	Hypothetical protein
13	Os10g0142600	2591399..2596634	3.20	Putative wall-associated protein kinase
14	Os10g0142700	2573722..2575920	3.05	Putative wall-associated protein kinase (non-protein coding RNA)
15	Os08g0193600	5457436..5458183	2.72	F-box protein-like
16	Os01g0204900	5756471..5757587	2.44	Hypothetical protein
17	Os01g0605500	23850865..23854747	−5.39	Kinesin-like protein KIF19
18	Os04g0116800	984402..985981	−5.90	Hypothetical protein
19	Os04g0103800	250445..251491	−8.59	WASH complex subunit CCDC53 homolog
20	Os08g0472600	23273330..23281518	−42.45	Glycoprotein 3-alpha-l-fucosyltransferase A

* Hygromycin gene (GenBank accession number, AF234299) [49].

## Supplementary Materials

Supplementary materials can be found at http://www.mdpi.com/1422-0067/19/4/1225/s1.

## Abbreviations

OsFucT	α1,3-fucosyltransferase
MALDI-TOF	Matrix-assisted laser desorption/ionization time-of-flight
ORF	Open reading frame
HM	Homozygote
HT	Heterozygote
SEM	Scanning electron microscopy
GEO	Gene Expression Omnibus
