# Supplementary material for: Lack of the α1,3-Fucosyltransferase Gene (Osfuct) Affects Anther Development and Pollen Viability in Rice
Source: Int J Mol Sci. 2018 Apr 18;19(4):1225. doi: 10.3390/ijms19041225 (PMC5979348; doi:10.3390/ijms19041225)
Supplement: Supplementary file 1 [file ijms-19-01225-s001.pdf]

Table S1. Primers used in this study.

Primer name	Primer sequence (5′ to 3′)	Associated gene region	Use	Expected amplified product size
**P1**	5′-GAGGCTTACGTGGGATTTC-3′ (23265977–23265997)	*Osfuct* genomic DNA	Genotyping	1,220 bp
**P2**	5′-GCAGATTTTACACAACATTTCGAC-3′ (23266215–23266239)	*Osfuct* genomic DNA	Genotyping	
**P3**	5′-GCATAAAGCTTGAGGGCAAG-3′ (23267177–23267197)	*Osfuct* genomic DNA	Genotyping	500 bp
**P4**	5′-ACGTCCGCAATGTGTTATTAAGTTGT-3′ (8212–8237)	*T-DNA*	Genotyping	
**P5**	5′-ACGACCAGGTCGTCTAACGACTCCG-3′ (23265083–23266007)	*Osfuct* cDNA/genomic DNA	RT-PCR/ cloning of genomic DNA	7,605 bp for gDNA & 1,542 bp for cDNA
**P6**	5′-AACGATATTGCTGTGATATAATGTAATGTACTATGTTATAACTG-3′ (23278263–23278307)			
**P7**	5′-GGTCCGCAGCATGCAAAGGC-3′ (23263321–23263341)	*Osfuct* promoter	Cloning	2,852 bp
**P8**	5′-GGGAGTTGGCAAGGCGACCTC-3′ (23265061–23265082)			
***Actin1* - F**	5′-CAACACCCCTGCTATGTACG-3′ (401–420)	*Actin1* cDNA	RT-PCR	508 bp
***Actin1* - R**	5′-GTTGCCATATAGATCCTTCC-3′ (889–898)			

Table S2. Primers used in the qRT-PCR analysis.

Primer name	Primer sequence (5′ to 3′)	Gene name	Expected amplified product size
Os03g0629800_S1F	5′-GCTCGTGGTGCATGATGATA-3′	Hypothetical protein	98 bp
Os03g0629800_S1R	5′-GGGAAGAGAAATCCTGTGAATCT-3′		
Os07g0162450_S1F	5′-GAGCAATCTCTGCTCCTCATATC-3′	Hypothetical protein	98 bp
Os07g0162450_S1R	5′-GTTTGTTGTCGAGCCGTAGA-3′		
Os01g0965300_S1F	5′-GTCGGAGATCGAGCGGTA-3′	Hypothetical protein	93 bp
Os01g0965300_S1R	5′-TCGACGACGACCACCTC-3′		
Os03g0299700_S1F	5′-TGGTTACGTTCAAGCGAAGAG-3′	Hypothetical protein	100 bp
Os03g0299700_S1R	5′-CGGCACCAGCATAAGAAGAA-3′		
Os03g0105500_S1F	5′-TGAATTCCTCGGTTGGATCTG-3′	Hypothetical protein	106 bp
Os03g0105500_S1R	5′-TCCTTGTCACTATGTTGCTCTT-3′		
Os07g0529000_S1F	5′-GGGCAAAGGAGTTACTGAAGAG-3′	Isocitrate lyase	89 bp
Os07g0529000_S1R	5′-TCCTGGATTTGGCAAGAACAT-3′		
Os01g0872900_S2F	5′-CGTTGGGAGAAGTGTCCTTTAG-3′	Glutathione S-transferase T3	99 bp
Os01g0872900_S2R	5′-CAAGTCCTGGGAAGCAACA-3′		
Os07g0511400_S1F	5′-GTTGGTGTCGGCGTTGG-3′	Hypothetical protein	90 bp
Os07g0511400_S1R	5′-CGCCACGTCGATGTCTTAAC-3′		
Os01g0698800_S1F	5′-CACTTCTCTGGCCGTCTTATT-3′	Hypothetical protein	93 bp
Os01g0698800_S1R	5′-GATCACCTAGAGAACGGGAAAC-3′		
Os10g0142700_S3F	5′-GCAGTATCCTCCGTCGTC-3′	Putative wall-associated protein	93 bp

Os10g0142700_S3R	5′-TTTGGTTGGCACCTCTTTC-3′	kinase (Non protein coding RNA)	
Os04g0301500_S1F	5′-TCATCACCGCCAACATCAC-3′	Transcription factor bHLH35	102 bp
Os04g0301500_S1R	5′-CCTCCACCATCTGCTTCATTT-3′		
Os10g0142600_S1F	5′-GCTAACAAAGGGAGCCTCTAC-3′	Putative wall-associated protein kinase (protein kinase APK1A, chloroplastic)	91 bp
Os10g0142600_S1R	5′-CCGACCCAACAGCAATATCT-3′		
Os01g0204900_S1F	5′-CGACTTGAGCTGCAAACTTATC-3′	Hypothetical protein	106 bp
Os01g0204900_S1R	5′-GTATTATCCATTGGGCCACATATTC-3′		
Os02g0441000_S1F	5′-GGAGAAGAGAGTGGTGGTTTC-3′	Hypothetical protein	102 bp
Os02g0441000_S1R	5′-CCACCTCCTAGCTGCAATAAT-3′		
Os11g0477400_S1F	5′-GAGAGGTGTCCGTGTGTTTAG-3′	Hypothetical protein	96 bp
Os11g0477400_S1R	5′-CCAGACTTGTTGAGCGTAACT-3′		
Os05g0211800_S1F	5′-TTAGCGAGGTTGCACCATATTA-3′	Hypothetical protein	101 bp
Os05g0211800_S1R	5′-GGCAAACACGAAGAATCAATACA-3′		
Os08g0193600_S3F	5′-GGGAATCAGGCGGAGATG-3′	F-box protein-like	97 bp
Os08g0193600_S3R	5′-GAGGGTTAGGGTTTGAGAGAG-3′		
Os09g0358000_S1F	5′-CACAGCTGAAGGAGAGTCTAAC-3′	Probable LRR receptor-like serine/threonine-protein kinase At1g51810	90 bp
Os09g0358000_S1R	5′-CCATAGCCACCACTACATCATC-3′		
Os12g0140266_S1F	5′-CAGTTCTACTGTGTGTGGAGAG-3′	Transcription initiation factor IIE subunit alpha isoform X1	91 bp
Os12g0140266_S1R	5′-GTCTTCCTGCCTCTTCTGTATG-3′		
Os02g0146600_S4F	5′-GAGAACTACCTCCATCGCATTG-3′	Eukaryotic initiation factor 4A-3	97 bp
Os02g0146600_S4R	5′-CGAACAGCATCCTCTCATCATC-3′		
Os02g0252400_S4F	5′-TACTACGCGCCTCTCATCA-3′	Dof zinc finger protein	88 bp

Os02g0252400_S4R	5′-GCTGCATCAGAGAACCAGAA-3′		
Os01g0137150_S3F	5′-CCAACTGTACCTCTTGCTTCA-3′	Hypothetical protein	101 bp
Os01g0137150_S3R	5′-GATGAGGACTTCTGCCCTAAAG-3′		
Os04g0192200_S4F	5′-CCTCACCATGCTCTCCAAA-3′	Hypothetical protein	77 bp
Os04g0192200_S4R	5′-AGAAGACGGTGGTCGATCT-3′		
Os02g0269650_S1F	5′-CGACCGGCCAACAAGAA-3′	Hypothetical protein	75 bp
Os02g0269650_S1R	5′-GTTGGAAATGGAGGAGGAGAAA-3′		
Os10g0340100_S3F	5′-TCAGATCCAACATCAACCTTCTC-3′	Hypothetical protein	89 bp
Os10g0340100_S3R	5′-TTTCCTCCCTCCACTCCAT-3′		
Os04g0397800_S1F	5′-CAGGCTAGGGTTTGGGATATTT-3′	Hypothetical protein	90 bp
Os04g0397800_S1R	5′-TCGCATGCGGTTGCTTAT-3′		
Os12g0543800_S3F	5′-AAGAAGACCGCCAATGATTCT-3′	Hypothetical protein	99 bp
Os12g0543800_S3R	5′-TCTTCCCTCACCTACTGTATTCT-3′		
Os09g0356800_S4F	5′-GCTGGATCACCGAGAATAGTG-3′	Probable LRR receptor-like serine/threonine-protein kinase At1g51810	99 bp
Os09g0356800_S4R	5′-TGATGTGACCATGACCTTGTAATA-3′		
Os04g0112100_S2F	5′-GGCTGGACAAGTCGATGAAT-3′	Putative disease resistance protein RGA4	98 bp
Os04g0112100_S2R	5′-ATCTCCTCCTCCTTGTGATGA-3′		
Os12g0169300_S4F	5′-AAGAAGACCGCCAATGATTCT-3′	Disheveled-associated activator of morphogenesis 1	99 bp
Os12g0169300_S4R	5′-TCTTCCCTCACCTACTGTATTCT-3′		
Os12g0228500_S1F	5′-AAGAAGACCGCCAATGATTCT-3′	Hypothetical protein	103 bp
Os12g0228500_S1R	5′-TCTTCCCTCACCTACTGTATTCT-3′		
Os01g0605500_S1F	5′-TGTTGCTGCTGGTCTTACTC-3′	Kinesin-like protein KIF19	99 bp
Os01g0605500_S1R	5′-CGGGTTGGTTCTGTTGTTCTA-3′		

Os04g0116800_S4F	5′-GGACATCATCAGTGAGCTTCAG-3′	Hypothetical protein	101 bp
Os04g0116800_S4R	5′-CACTACCATCATTGTACTACTCCTATTT-3′		
Os04g0103800_S3F	5′-CTCACTCCTCTCCCACGTC-3′	WASH complex subunit CCDC53 homolog	97 bp
Os04g0103800_S3R	5′-TCGTCGTCGCTCGTCAT-3′		
Os01g0974600_S4F	5′-GCTGGTGGACATTTATCCCATTA-3′	Glycine-rich RNA-binding protein 2	78 bp
Os01g0974600_S4R	5′-GAGGCAGAAGCAATCCAACT-3′		
Os04g0125700_S2F	5′-CGTACTCTTGGACGACGATTT-3′	L-type lectin-domain containing receptor kinase IX.1	101 bp
Os04g0125700_S2R	5′-AGCCTATGGCAGTTGTCTTC-3′		
Os01g0629000_S2F	5′-GCACTCTGCAGGCAAAGATA-3′	Hypothetical protein	79 bp
Os01g0629000_S2R	5′-TGCAGAATCTGAAGACTGTTGT-3′		
Os03g0794000_S1F	5′-ACCACTCCAGCATCCACTA-3′	Non-specific lipid transfer protein GPI-anchored 2 isoform X1	77 bp
Os03g0794000_S1R	5′-GCGACGAGGATGACCAATATC-3′		
Os09g0286300_S2F	5′-CCATGATTGGAGGGAGGAAAG-3′	ENDOSPERM DEFECTIVE 1	89 bp
Os09g0286300_S2R	5′-GAACCTGTACTGGAGGTAGGA-3′		
Os10g0552600_S1F	5′-CTCTGCACCGCCATCAA-3′	Cortical cell-delineating protein	83 bp
Os10g0552600_S1R	5′-CCGCAGTTGTTGAGGATGA-3′		
Os08g0472600_S1F	5′-GTTCCTGTGTGGAAGGATGAA-3′	Glycoprotein 3-alpha-L-fucosyltransferase A	89 bp
Os08g0472600_S1R	5′-TGTGTAAGGCCGATTGGATAAA-3′		
Hygromycin_S1F	5′- GCTTTCAGCTTCGATGTAGGA -3′	Hygromycin	96 bp
Hygromycin_S1R	5′- CGATGCAAAGTGCCGATAAAC -3′		
UBI_S1F	5′- GAAGTAAGGAAGGAGGAGGA -3′	Ubiquitin	99 bp
UBI_S1R	5′- AAGGTGTTCAGTTCCAAGG -3′		

Table S3. Structures and corresponding calculated masses of the major N-glycans for the wild-type (DJ), *Osfuct* mutant (HM, homozygote; HT, heterozygote) and restore lines (R12) plants.

m/z	Structure	DJ	HM	HT	R12
1065.2	$Man_3XylGlcNAc_2$	+	+	+	+
1081.2	$Man_3FucGlcNAc_2$	+	+	+	+
1211.3	$Man_3XylFucGlcNAc_2$	+	-	+	+
1268.7	$GlcNAcMan_3XylGlcNAc_2$	-	+	+	+
1414.6	$GlcNAcMan_3XylFucGlcNAc_2$	+	-	+	+
1617.4	$GlcNAc_2Man_3XylFucGlcNAc_2$	+	-	+	+
1926.3	(FA)GnFXF/Gn(FA)XF	+	-	+	+
2235.6	$(FA)(FA)XF_3$	+	-	+	+

A calculated mass of the major N-glycans was compared with earlier reports to assign the corresponding structures ([15], [16], [40]). A, galactose; F, fucose; (FA), Lewis A determinant; Gn, *N*-acetylglucosamine; M, mannose; X, xylose. For the

corresponding structures, refer to **Figure S3**.

Table S4. Microarrays genes significantly (log_2 fold change> 1.6and *p*-value < 0.1) up-regulated in *Osfuct* mutant compared with wild-type (Dongjin).

SN	Gene ID	Gene location	DJ vs HM (log_2 ratio)	Gene descriptions
1	Os07g0162450	3371536..3372781	21.91	Hypothetical protein
2	Os07g0529000	20691103..20693734	10.46	Isocitrate lyase
3	Os12g0169300	3525459..3528156	4.53	Disheveled-associated activator of morphogenesis 1
4	Os12g0228500	6984194..6988897	4.47	Hypothetical protein
5	Os01g0872900	37860129..37865917	3.74	Glutathione S-transferase T3

6	Os01g0698800	28910211..28911075	3.04	Hypothetical protein
7	Os04g0301500	13466445..13468863	2.95	Transcription factor bHLH35
8	Os05g0211800	6904022..6904580	2.67	Hypothetical protein
9	Os11g0477400	16708756..16734818	2.54	Hypothetical protein
10	Os02g0441000	14947871..14950082	2.49	Hypothetical protein
11	Os02g0252400	8590287..8594087	2.12	Dof zinc finger protein
12	Os12g0140266	1942163..1948013	1.98	Transcription initiation factor IIE subunit alpha isoform X1
13	Os09g0358000	11576022..11588924	1.93	Probable LRR receptor-like serine/threonine-protein kinase At1g51810
14	Os02g0146600	2557564..2561015	1.83	Eukaryotic initiation factor 4A-3
15	Os01g0137150	1991244..1996867	1.69	Hypothetical protein
16	Os04g0192200	6265896..6271636	1.67	Hypothetical protein

Table S5. Microarrays genes significantly ($\log_2$ fold change> 1.6 and *p*-value < 0.1) down-regulated in *Osfuct* mutant compared with wild-type (Dongjin).

**SN**	**Gene ID**	**Gene location**	**DJ vs HM ($\log_2$ ratio)**	**Gene descriptions**

1	Os10g0552600	21690306..21697772	-2.18	Cortical cell-delineating protein
2	Os09g0286300	6368292..6371219	-1.95	ENDOSPERM DEFECTIVE 1
3	Os01g0974600	43070805..43071131	-1.82	Glycine-rich RNA-binding protein 2
4	Os04g0125700	1562256..1572689	-1.81	L-type lectin-domain containing receptor kinase IX.1
5	Os01g0629000	26781929..26784102	-1.74	Hypothetical protein
6	Os03g0794000	33027845..33029314	-1.65	Non-specific lipid transfer protein GPI-anchored 2 isoform X1

Table S6. Experimental design of Roche Nimblegen *Oryza sativa* 135 K microarray.

No.	Sample	Type	Comment
1	Dongjin_1	Control	Cultivar to make *Osfuct* mutant
2	Dongjin_7	Control	Cultivar to make *Osfuct* mutant
3	Dongjin_8	Control	Cultivar to make *Osfuct* mutant
4	PMHMABFT5	*Osfuct* mutant	Homozygote of *Osfuct* mutant
5	PMHMABFT8	*Osfuct* mutant	Homozygote of *Osfuct* mutant
6	PMHMABFT9	*Osfuct* mutant	Homozygote of *Osfuct* mutant

Table S7. Experimental metrics report.

IMAGE_	INTER	SIGNAL	UNIFOR	UNIFOR	NUM_	MEAN_	NUM_EX	MEAN_	NUM_R	MEAN_R

NAME	QUARTILE_DENSITY	_RANGE	MITY_MEAN	MITY_CV	EMPTY	EMPTY	PERIMENTAL	EXPERIMENTAL	ANDOM	ANDOM
Dongjin1	2.81	0.239	3044.05	0.05	146232	826.06	137604	2779	11735	390.03
Dongjin7	2.90	0.38	3664.08	0.07	146232	910.46	137604	3335	11735	409.48
Dongjin8	2.99	0.33	3996.99	0.06	146232	933.00	137604	3558	11735	381.90
PM-HM-ABFT5	2.93	0.52	3914.91	0.08	146232	920.90	137604	3496	11735	407.02
PM-HM-ABFT8	2.87	0.25	3413.38	0.05	146232	910.88	137604	3122	11735	404.10
PM-HM-ABFT9_	2.87	0.27	4105.82	0.04	146232	1036.66	137604	3725	11735	465.37

IMAGE_NAME (the name of the analyzed image file); INTERQUARTILE_DENSITY (the interquartile range of the raw signal intensities); SIGNAL_RANGE (the signal range represents the signal range on a per channel basis for both one- and two-color microarray applications); UNIFORMITY_MEAN (the mean signal intensity of all the probes in each uniformity block); UNIFORMITY_CV (the coefficient of variation of the block uniformity means); NUM_EMPTY (the number of empty features present on the array); MEAN_EMPTY (the mean signal intensity of empty features present on the array); NUM_EXPERIMENTAL (the number of experimental features present on the array); MEAN_EXPERIMENTAL (the mean signal intensity of the experimental features present on the array); NUM_RANDOM (the number of random control features present on the array); MEAN_RANDOM (the mean signal intensity of the random control features present on the array). Reference: NimbleGen Arrays User's Guide: CGH Analysis v6.0

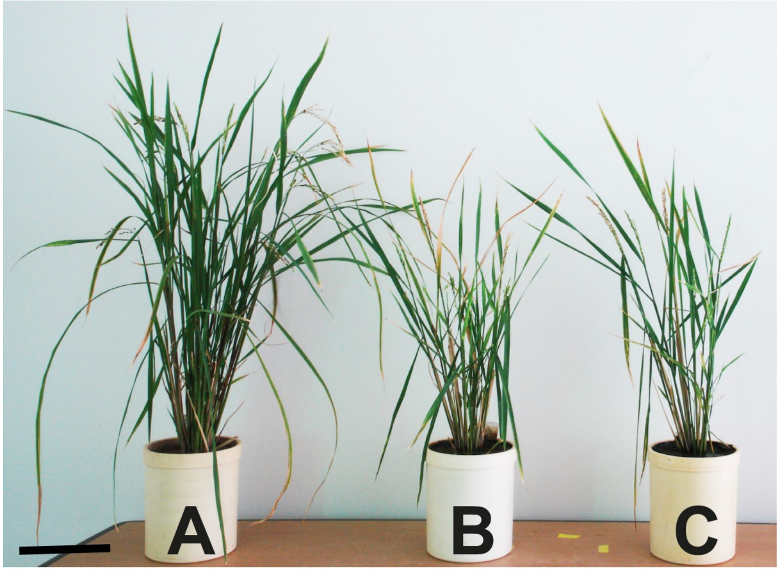

Figure S1. Phenotype of *Osfuct* mutant. Morphological phenotype of mutant (HM and HT) compared with Dongjin. **(A)** Phenotype of Dongjin (**B)** HM. **(C)** HT. Scale bar is 17 cm.

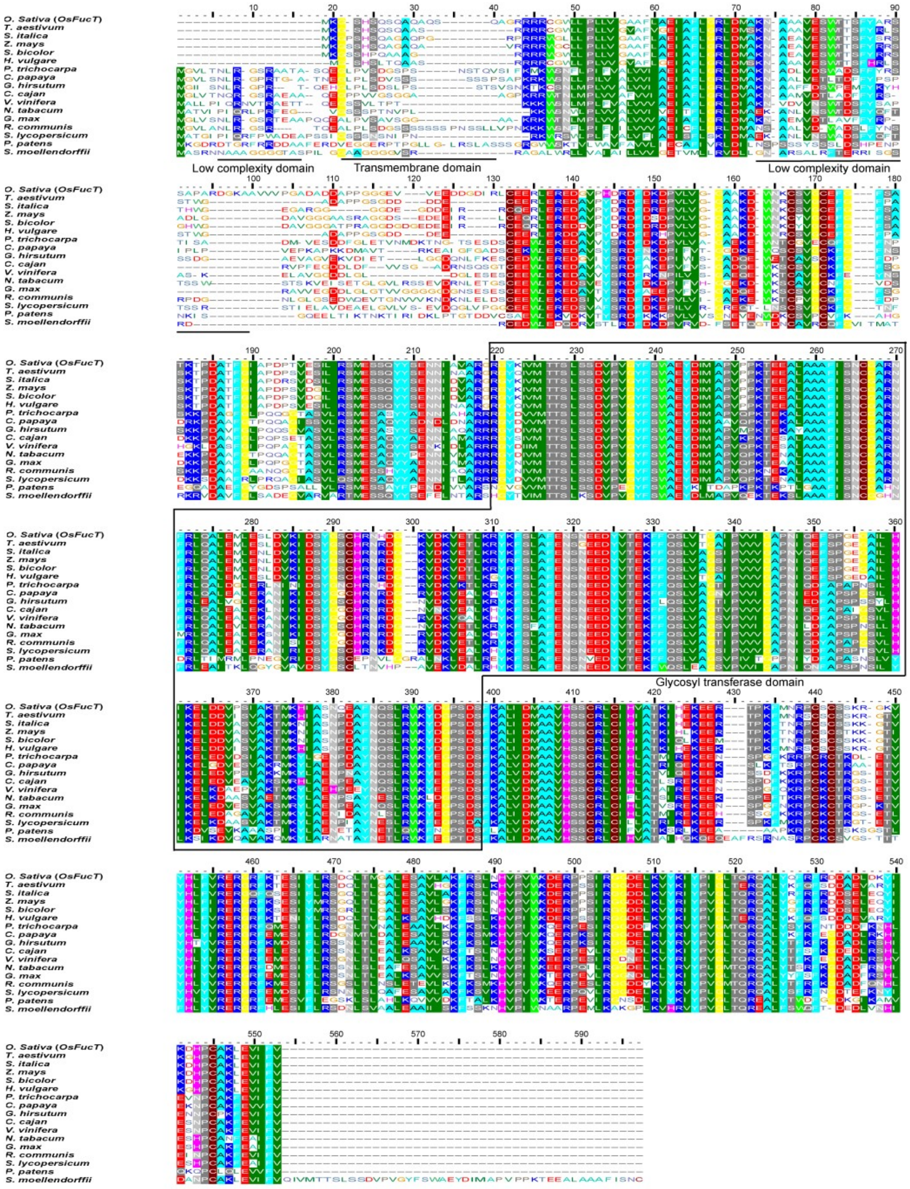

Figure S2. Multiple sequence alignment of OsFucT with other glycosyltransferase proteins. Comparisons of the deduced amino sequence of OsFucT with glycosyl transferase proteins from *T. aestivum, H. vulgare, S. italica, Z. mays, S. bicolor, C. cajan, G. max, N. tabacum, S. lycopersicum, S. moekkendorffii* and *P. patens*. A number of the amino acid residue is shown in the upper of the sequence. Conserved glycosyl transferase domain shown in the black box, low complexity and transmembrane domain is underlined. Chemically similar residues are denoted with a color and

shade. Dashes indicate gaps introduced to maximize the alignment of homologous region.

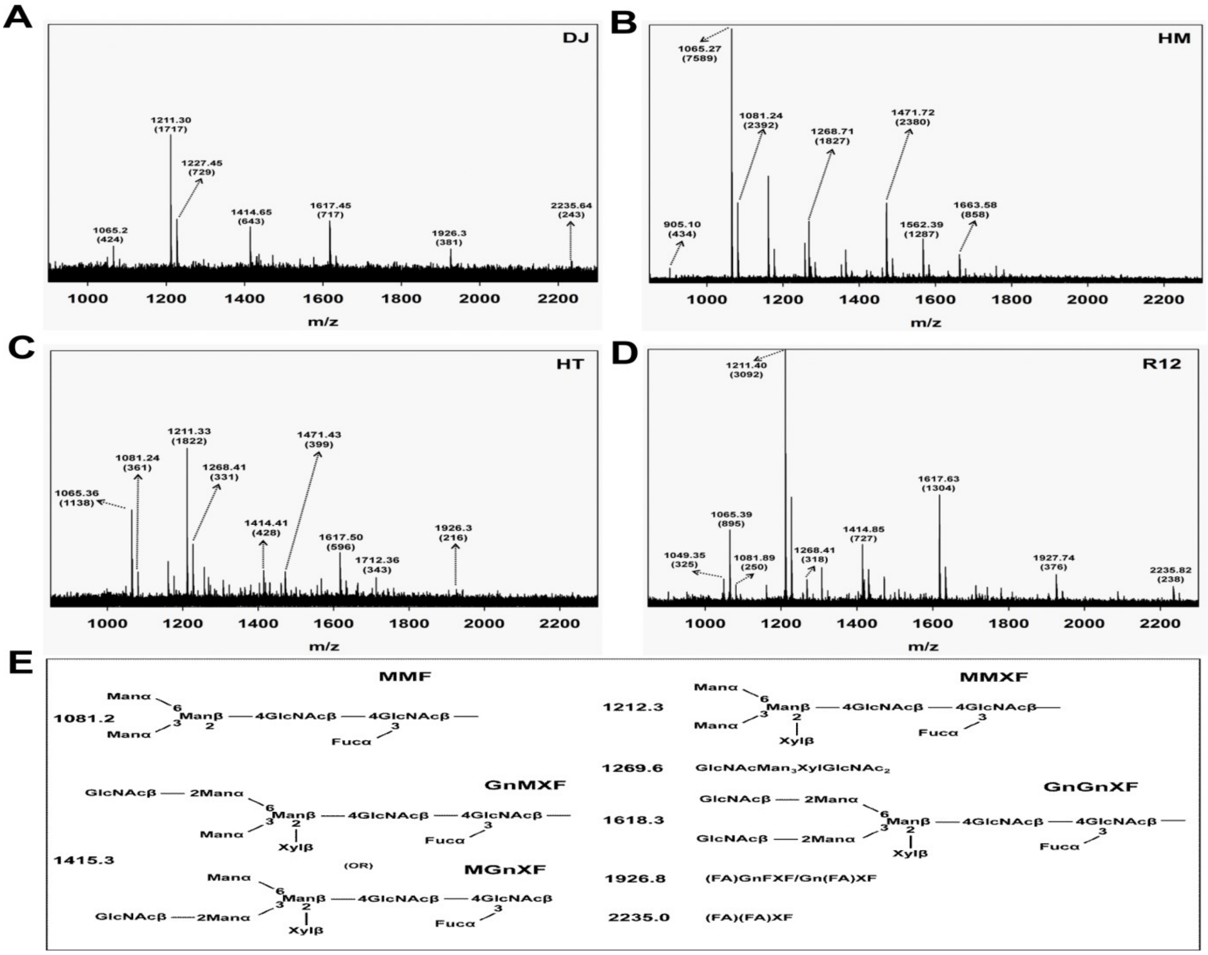

Figure S3. Glycans profiling of Dongjin (wild-type), *Osfuct* mutant (HM and HT) and rescued rice line using mass spectrometry (MALDI-TOF). **(A)** Dongjin; **(B)** HM, homozygote line; **(C)** HT, heterozygote line; **(D)** R12, rescued lines. Values in parentheses indicate numerical representation of the peak intensity. **(E)** Representative structures examples of *N*-linked glycans obtained from Dongjin,

mutant (HM, homozygous; HT, heterozygous) and rescued line (R12). M, mannose; F, fucose; X, xylose; Gn, *N*-acetylglucosamine; A, galactose; (FA), Lewis A determinant ([15], [16], [40]).

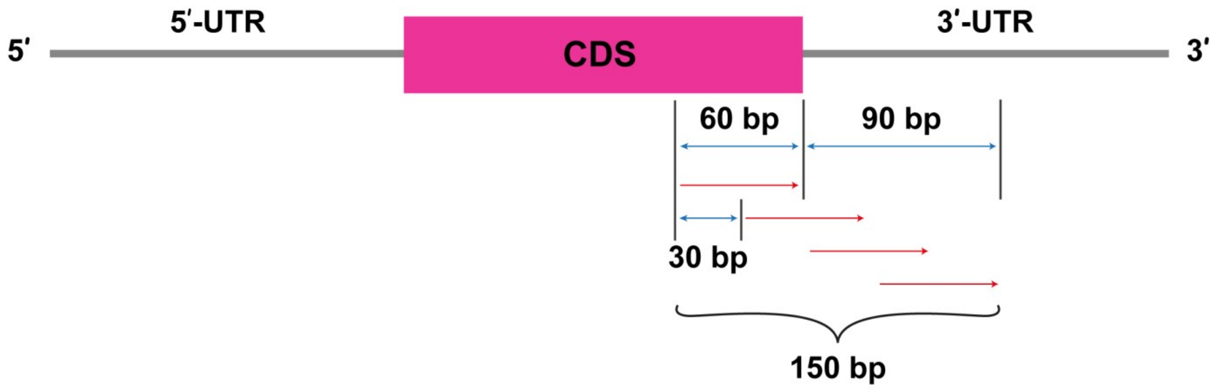

Figure S4. Chip information of whole genome 135K oligo microarray. Sequence source (RAP2), probe length (60 mer), probe per array (125,956), feature size (13 μm x 13 μm), array dimensions (8.9 mm x 6.5 mm), overall slide dimensions (25 x 75 mm).

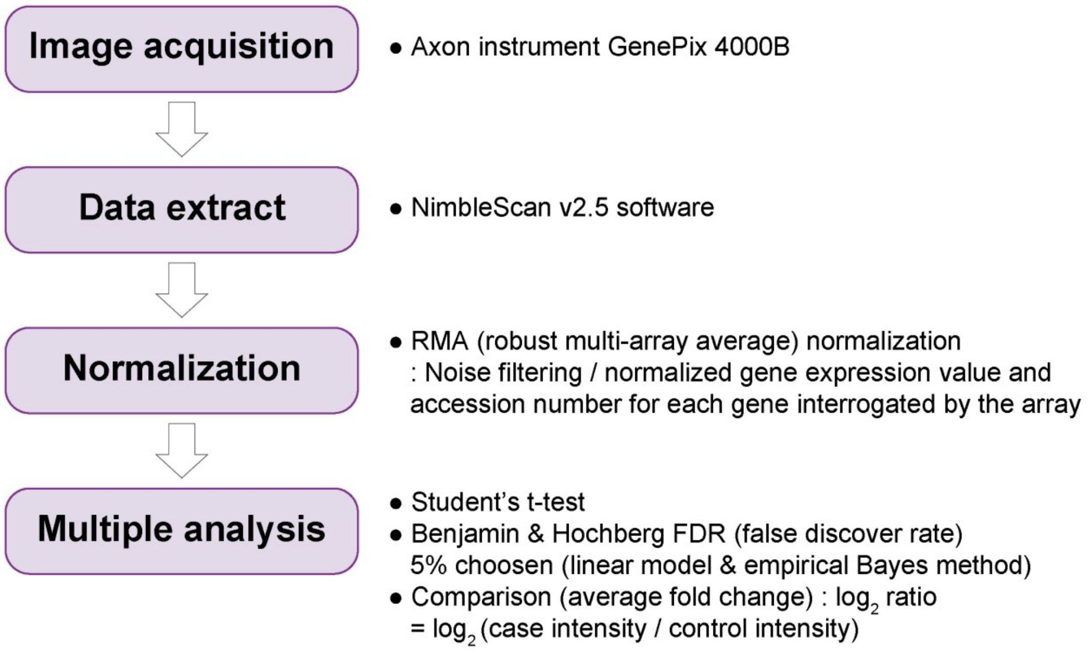

Figure S5. Scheme of analysis of expression microarray data.

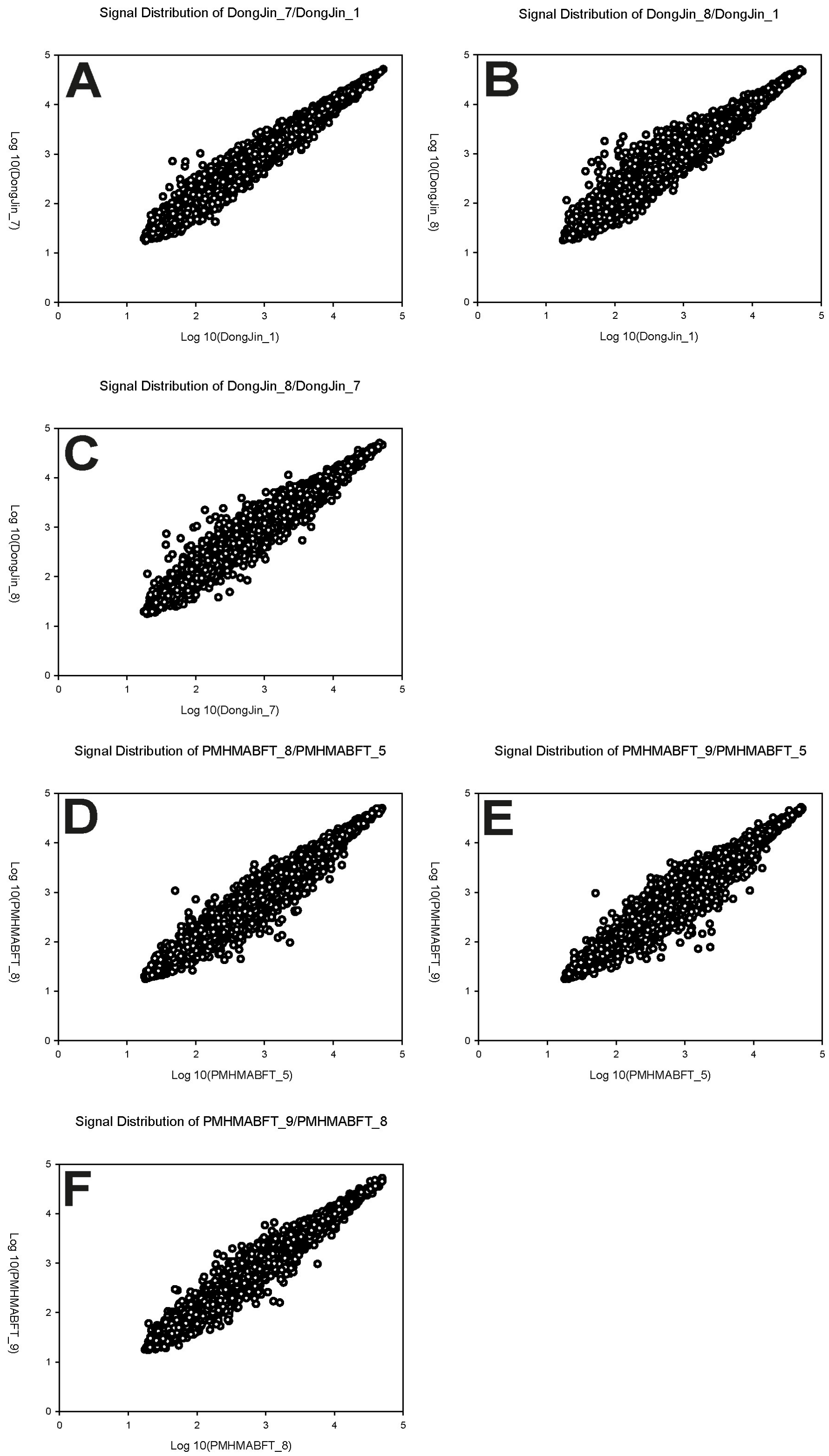

Figure S6. Signal distribution in Dongjin and PH_HM_ABF microarrays. Signal comparison between Dongin **(A-C)** and PH_HM_ABF microarrays **(D-F)**.

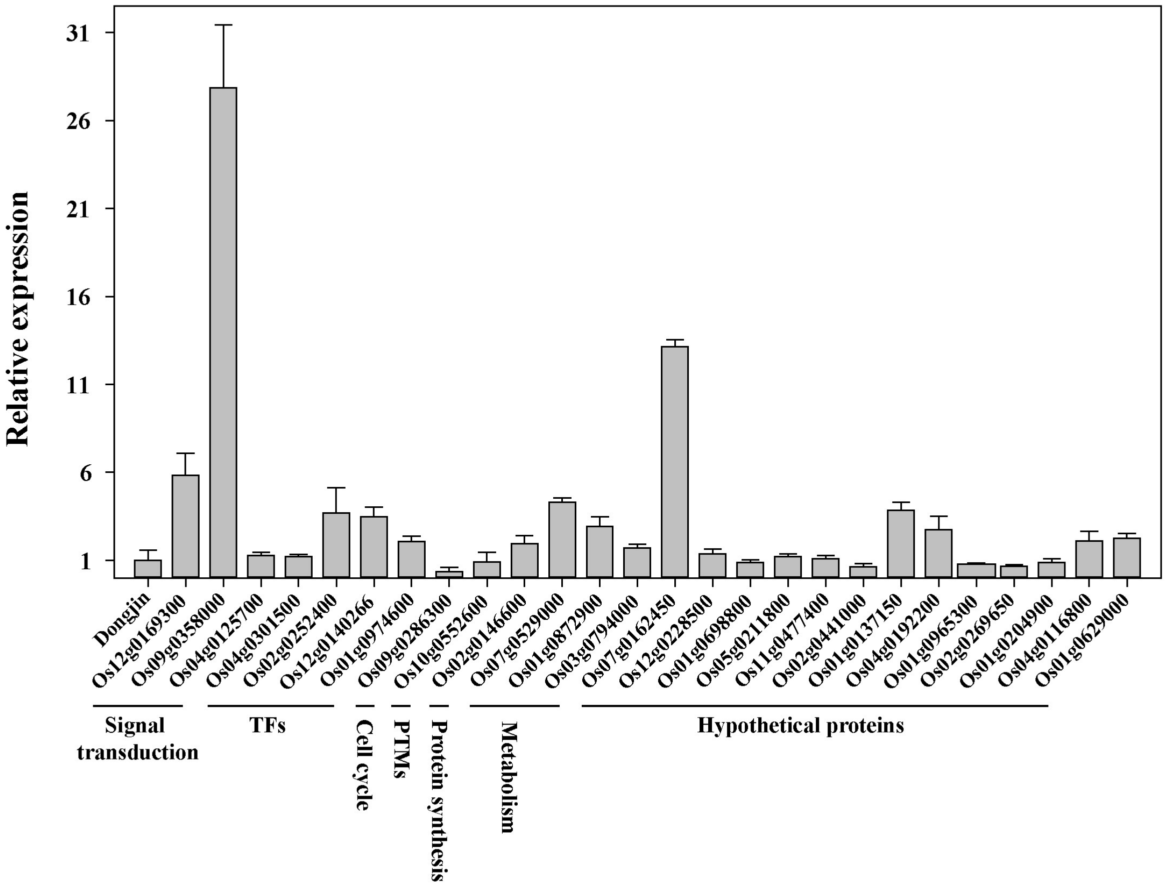

Figure S7. qRT-PCR results for the differentially expressed transcripts in wild-type (DJ) and *Osfuct* mutant (HM) to verify transcriptome profile data produced by microarray analysis. Error bars show standard deviation.
